# Weakly Polar Organic Additive Inducing Capacity‐Dependent Zinc Growth Transition via Indirect Solvation and Adsorption Engineering in Aqueous Electrolytes

**DOI:** 10.1002/smtd.202502427

**Published:** 2026-03-19

**Authors:** Sung‐Ho Huh, Beom‐Keun Cho, Yao‐Peng Chen, So Hee Kim, Jong‐Seong Bae, Xiang Chen, Seung‐Ho Yu

**Affiliations:** ^1^ Department of Chemical and Biological Engineering Korea University Seoul Republic of Korea; ^2^ Beijing Key Laboratory of Complex Solid State Batteries & Tsinghua Center for Green Chemical Engineering Electrification Department of Chemical Engineering Tsinghua University Beijing P. R. China; ^3^ The Innovation Center for Smart Solid State Batteries Yibin P. R. China; ^4^ Advanced Analysis Center Korea Institute of Science and Technology (KIST) Seoul Republic of Korea; ^5^ Yeongnam Regional Center Korea Basic Science Institute (KBSI) Busan Republic of Korea; ^6^ Department of Battery‐Smart Factory Korea University Seoul Republic of Korea

**Keywords:** aqueous electrolytes, corrosion inhibition, electrolyte additives, zinc deposition, zinc metal anodes

## Abstract

Aqueous zinc batteries have emerged as promising candidates for safe and sustainable energy storage. However, their practical application is severely limited by zinc corrosion, hydrogen evolution, and non‐uniform dendritic growth stemming from interfacial instability and water‐induced side reactions. Herein, we report dimethyl isosorbide (DMI) as an effective electrolyte additive that simultaneously regulates zinc ion solvation structure and stabilizes the zinc/electrolyte interface. DMI modulates the solvation shell by restructuring the hydrogen‐bonding network while adsorbing onto zinc surfaces to form a protective molecular layer. Comprehensive spectroscopic analyses and molecular dynamics simulations reveal weakened zinc solvation power and reduced H_2_O activity in the presence of DMI, leading to suppression of zinc corrosion. Notably, DMI induces a capacity‐dependent crystallographic zinc evolution, enabling a transition from preferential initial growth to stable deposition at higher areal capacities. Electrochemical evaluations demonstrate prolonged cycling stability, near‐unity Coulombic efficiency, and robust performance under high current density and high areal capacity conditions. *Operando* optical visualization and morphology analyses confirm highly uniform, dendrite‐free zinc deposition and nearly reversible zinc plating/stripping. This work highlights an effective electrolyte engineering strategy for stabilizing zinc metal anodes and advancing the practical viability of aqueous zinc batteries.

## Graphical abstract

Dimethyl isosorbide (DMI) regulates Zn solvation and stabilizes the Zn/electrolyte interface, suppressing corrosion, ZBS, and dendrites, enabling uniform plating and long‐life, high‐rate aqueous zinc batteries.

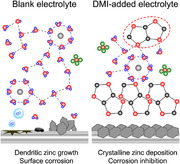

## 1 Introduction

The rapid expansion of electric vehicles, grid‐scale energy storage systems, and portable electronic devices has driven an unprecedented demand for high‐performance rechargeable batteries [1]. Among the available energy storage technologies, lithium (Li)‐ion batteries (LIBs) have dominated the market owing to their high energy density, long cycle life, and well‐established manufacturing infrastructure. However, the increasing demands for safety, cost‐effectiveness, and sustainability have highlighted the intrinsic limitations of conventional LIB systems. In particular, the inherent flammability of organic electrolytes poses significant safety risks, including electrolyte leakage, thermal runaway, and the risk of fire or explosion under abusive conditions [2, 3]. These issues become more critical as battery energy density increases and large‐format cells are widely deployed in electric vehicles and stationary energy storage systems. In addition, the dependence on limited resources and complex cell architectures further exacerbates concerns related to cost and environmental impact. Consequently, the development of safe and sustainable battery systems has emerged as a key priority for next‐generation energy storage technologies [4, 5].

In this context, aqueous electrolyte batteries employing zinc metal anodes have attracted considerable attention as promising alternatives due to their intrinsic safety, non‐flammability, low cost, and environmental friendliness [6]. The use of water‐based electrolytes effectively eliminates combustion risks while providing high ionic conductivity, enabling fast charge–discharge kinetics and high‐power density [7]. Moreover, zinc metal is considered a highly attractive anode material because of its high theoretical specific capacity (820 mAh g^−1^) and relatively low redox potential (−0.76 V vs. standard hydrogen electrode). These advantages render aqueous zinc batteries particularly suitable for large‐scale and safety‐critical energy storage applications. Nevertheless, their practical implementation remains hindered by fundamental challenges, including the narrow electrochemical stability window of water and severe interfacial instability at the zinc metal anode. Undesirable side reactions, such as hydrogen evolution reaction (HER), zinc corrosion, and non‐uniform zinc dendrite growth, significantly degrade cycling stability and Coulombic efficiency (CE). Importantly, the degradation processes in aqueous zinc batteries are not independent but are strongly coupled, leading to progressive performance deterioration [8, 9]. For example, water electrolysis at the electrode surface induces hydrogen evolution, which locally increases hydroxide ion concentration and generates an alkaline microenvironment. Under such conditions, Zn^2+^ readily participates in side reactions with sulfate ions (SO_4_^2−^), resulting in the formation of zinc basic sulfate (ZBS) on the electrode surface. The ZBS layer acts as an electrochemically inactive passivation film that impedes zinc plating and stripping. Therefore, the effective electrochemically active surface area of the zinc anode decreases, increasing the local current density at the remaining active sites and thereby accelerating dendritic zinc growth. The formation of zinc dendrites further enlarges the electrode surface area, which in turn promotes additional side reactions and water electrolysis. These mutually reinforcing processes establish a self‐amplifying degradation pathway that severely compromises long‐term battery performance. Therefore, effective mitigation of zinc anode failure requires integrated strategies that simultaneously address these interconnected phenomena rather than treating them individually.

Current strategies for improving zinc metal anode performance in aqueous zinc batteries can be broadly categorized into electrode engineering and electrolyte engineering approaches. Electrode engineering focuses on modifying the anode itself to suppress corrosion and promote uniform zinc deposition, including the introduction of zincophilic substrates and the construction of protective interfacial layers. Zincophilic metal strategies typically employ metals capable of forming alloys with zinc [10, 11, 12]. By synthesizing alloy anodes or introducing nucleation seeds on the anode surface, zinc plating and stripping can be partially replaced by alloying and dealloying reactions, thereby maintaining a more uniform electrode morphology during cycling while simultaneously reducing corrosion. These approaches can effectively enhance Coulombic efficiency and suppress the formation of dead zinc. Protective layer strategies, in contrast, rely on coating the zinc surface with porous materials, polymers, or carbon‐based layers to regulate Zn^2+^ transport, enable homogeneous zinc deposition, and inhibit interfacial corrosion [13, 14, 15, 16]. Despite their effectiveness, electrode engineering strategies also suffer from inherent limitations. Zincophilic metal anodes often involve electrochemically inert or less active components, which reduce the overall specific capacity, while the use of noble or rare metals undermines the cost advantages of aqueous zinc batteries. Protective layers may also suffer from mechanical degradation, such as cracking or delamination during prolonged cycling or under high areal capacity conditions, compromising long‐term stability.

Electrolyte engineering strategies, by contrast, modulate the interfacial behavior of the zinc anode by modifying the electrolyte composition, such as altering the solvent or salt chemistry or introducing functional additives [17, 18, 19]. In general, electrolyte engineering improves zinc anode performance through three primary mechanisms. First, specific molecules or ions can form adsorption layers or induce solid electrolyte interphase (SEI) formation on the zinc surface, thereby blocking direct contact between water molecules and the metal [20]. Second, electrolyte components can participate in or regulate the solvation structure of Zn^2+^, reducing the number of free water molecules involved in zinc deposition and suppressing both corrosion and hydrogen evolution [21]. Third, additives capable of forming hydrogen bonds with water molecules can decrease water activity at the electrode interface, further mitigating parasitic reactions.

Among electrolyte engineering approaches, additive‐based strategies have been extensively investigated because a wide range of water‐soluble compounds can be readily employed, and diverse functionalities can be achieved depending on the chemical nature of the additive [16, 22, 23]. In this study, dimethyl isosorbide (DMI), a derivative of the organic compound isosorbide, was selected as an electrolyte additive from various water‐soluble candidates. Unlike isosorbide, DMI is substituted with ─OCH_3_ groups, weakly interacting with Zn^2+^ or water molecules. Consequently, DMI indirectly regulates Zn^2+^ solvation structure by modulating the hydrogen‐bonding network of water molecules through the lone pairs of its four oxygen atoms. This indirect regulation of Zn^2+^ solvation structure was confirmed through both experimental characterization and theoretical calculations. In addition, the four oxygen atoms in DMI provide sufficient interaction strength with metallic zinc, enabling the formation of a molecular adsorption layer on the zinc surface. This interfacial molecular layer effectively suppresses surface corrosion and promotes uniform zinc deposition, ultimately leading to improved electrochemical performance in aqueous zinc batteries, as demonstrated in this work.

## 2 Results and Discussion

### 2.1 Characterization of DMI‐Added Electrolyte

**FIGURE 1:**
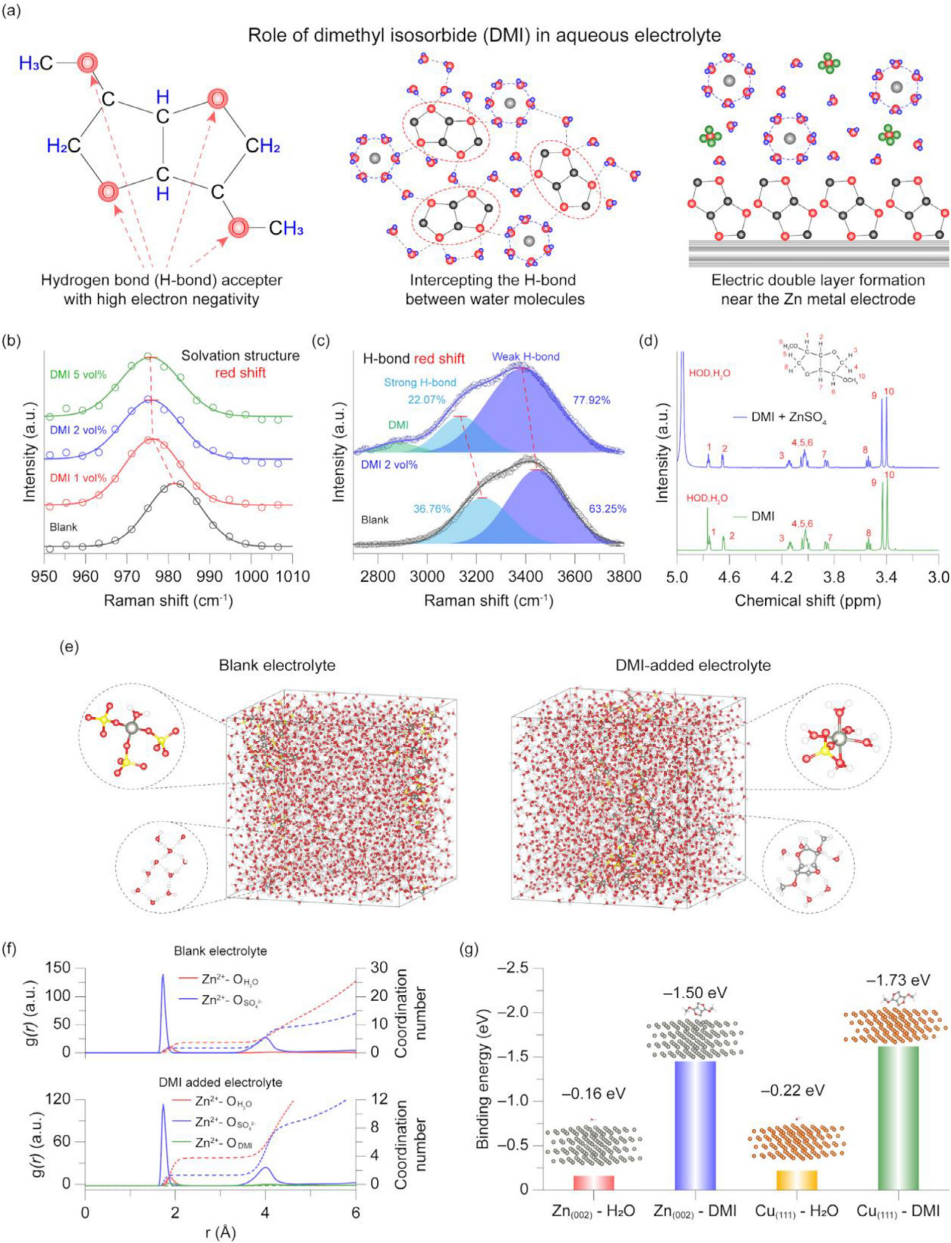
(a) Schematics illustrations of effects of DMI additive in aqueous electrolyte. (b) Raman spectra of the blank electrolyte and DMI‐added electrolytes within the Raman shift of 950–1005 cm^−1^. (c) Raman spectra of the blank electrolyte and DMI‐added electrolyte within the Raman shift of 2800–3800 cm^−1^. (d) ^1^H NMR spectra of the DMI 2 vol% solution and the DMI‐added electrolyte. (e) Snapshots of the electrolyte system and configuration of solvation structures of Zn^2+^ from MD simulations. In this visualization, zinc, oxygen, nitrogen, carbon, and hydrogen atoms are colored gray, red, black, and white, respectively. (f) Radial distribution functions for Zn^2+^ in the blank electrolyte and DMI‐added electrolyte. (g) The density functional theory (DFT) calculation results of H_2_O and DMI molecule adsorption energy on Zn(002) lattice and Cu(111) lattice.

The DMI‐added aqueous electrolyte was prepared through a simple process. Deionized water and DMI were first mixed at a fixed volume ratio to obtain a mixed solvent, which was then used to dissolve ZnSO_4_ to form a 1 m ZnSO_4_ electrolyte. The role of DMI in a ZnSO_4_‐based aqueous zinc electrolyte is illustrated (Figure 1a). DMI molecules contain four ether bonds, imparting weak polarity, and can interact with water molecules through the lone pairs of electrons on the oxygen atoms in the ether groups. Through these interactions, DMI can participate in or indirectly regulate the Zn^2+^ solvation environment, leading to a weakened solvation structure. Such regulation facilitates faster desolvation during zinc deposition while suppressing the generation of free water molecules at the electrode surface. In addition, owing to the presence of multiple oxygen atoms, DMI is expected to strongly adsorb onto metallic zinc surfaces, forming a molecular adsorption layer that modifies the electrode‐electrolyte interface.

Raman spectroscopy was employed to experimentally investigate the influence of DMI on the electrolyte structure. The Raman spectra in the range of 950–1010 cm^−1^ provide information on the Zn^2+^ solvation structure (Figure 1b) [24]. In the blank electrolyte without any additive, a characteristic solvation‐related peak appears at 981.55 cm^−1^. In contrast, electrolytes prepared using mixed solvents with different DMI volume ratios exhibit noticeable shifts in peak position. Specifically, the solvation‐related peak shifts to 976.5 cm^−1^ for DMI 1 vol%, 976.2 cm^−1^ for DMI 2 vol%, and 975.8 cm^−1^ for DMI 5 vol%, indicating a progressive red shift with increasing DMI content. These red shifts suggest a weakened Zn^2+^ solvation structure induced by the presence of DMI [25]. Raman spectra in the range of 2700–3800 cm^−1^ were further analyzed to probe the hydrogen‐bonding (H‐bond) network among water molecules (Figure 1c). In the blank 1 M ZnSO_4_ electrolyte, the fraction of strong H‐bonded water clusters (water molecules participating in multiple hydrogen bonds) is 22.07%, while weak H‐bonded clusters (water molecules involved in fewer hydrogen bonds) account for 77.93%. The corresponding Raman shifts for strong and weak H‐bonds are located at 3226.5 and 3448.3 cm^−1^, respectively. Upon introducing DMI 2 vol% into the electrolyte, both peaks exhibit red shifts (strong H‐bond: 3197 cm^−1^, weak H‐bond: 3424.1 cm^−1^), accompanied by a decrease in the fraction of strong H‐bonded water clusters. Such red shifts in the H‐bond‐related Raman peaks originate from strong interactions between water molecules and the oxygen atoms in DMI [26]. Moreover, the reduction in strong H‐bond content can be attributed to steric hindrance effects, whereby the bulky DMI molecules disrupt the formation of extensive hydrogen‐bonding networks among water molecules [27]. To verify whether steric hindrance induces changes in the hydrogen‐bonding clusters, molecular dynamics (MD) simulations were performed to quantify the number of hydrogen bonds per unit volume (Figure S1). The results show that, upon the addition of DMI to the electrolyte, the average hydrogen‐bond density decreases from 49.75 in the absence of DMI to 49.55 with DMI, confirming that DMI weakens the hydrogen‐bonding network among water molecules. To further examine structural changes in the electrolyte, Fourier‐transform infrared (FTIR) spectroscopy was performed (Figure S2). Although no obvious changes are observed in the overall spectra, magnified analysis reveals distinct DMI‐induced effects. In the expanded wavenumber range of 2700–3500 cm^−1^, a gradual red shift of the H─O stretching vibration is observed with increasing DMI content. This red shift indicates strong interactions between water molecules and DMI, confirming that DMI participates in the hydrogen‐bonding network of water [28]. These FTIR results are consistent with the trends observed in Raman spectroscopy. In addition, analysis of the expanded wavenumber ranges from 1800 to 800 cm^−^^1^ reveals the emergence of characteristic peaks associated with ether bonds and C─H bonds originating from DMI. Finally, to determine whether DMI directly participates in the Zn^2+^ solvation shell, ^1^H nuclear magnetic resonance (NMR) measurements were carried out (Figure 1d). Analysis of deuterated water with DMI 2 vol% shows well‐defined peaks corresponding to the hydrogen atoms of DMI. After dissolving ZnSO_4_ into the solution, the ^1^H NMR spectra reveal that the DMI‐related peaks remain unchanged, whereas the peak associated with water molecules exhibits a blue shift. The preservation of DMI‐related peaks indicates that DMI does not directly coordinate with Zn^2+^ in their primary solvation shell. Instead, indirect modulation through DMI‐water interactions affects the structure and strength of the Zn^2+^ solvation structure. This conclusion is consistent with the Raman results in Figure 1b, demonstrating that DMI alters the Zn^2+^ solvation structure indirectly by restructuring the hydrogen‐bond network of water molecules rather than through direct coordination with Zn^2+^.

To gain deeper insight into the DMI‐induced changes in electrolyte structure and electrode surface states, molecular dynamics simulations and adsorption energy calculations were performed [29, 30]. First, MD simulations were conducted to compare the Zn^2+^ solvation structures in a routine 1 m ZnSO_4_ blank electrolyte and DMI 2 vol% added electrolyte (Figure 1e). In the blank electrolyte, the fractions of solvation structures with H_2_O: SO_4_^2−^ ratios of 1: 3 and 5: 1 were determined to be 24.35% and 67.61%, respectively. In contrast, the incorporation of DMI shifted the fractions of these solvation structures to 17.07% for H_2_O: SO_4_^2−^ = 1: 3 and 74.60% for H_2_O: SO_4_^2−^ = 5: 1, indicating a clear reduction in the population of strong solvation structures involving multiple SO_4_^2−^ anions. To quantitatively evaluate the solvation structure modulation induced by DMI, radial distribution functions (RDFs) and coordination numbers (CNs) were analyzed (Figure 1f). In the blank electrolyte, the first solvation shell of Zn^2+^ consists of an average of 3.64 water molecules and 1.72 sulfate anions. Upon the introduction of DMI, the number of coordinating water molecules slightly increases to 3.95, while the number of SO_4_^2−^ decreases to 1.55, confirming the reduced participation of SO_4_^2−^ in the Zn^2+^ solvation shell. Furthermore, the number of DMI molecules participating in the first solvation shell is negligible (< 0.01), consistent with the ^1^H NMR results, indicating that DMI molecules rarely directly coordinate with Zn^2+^. This behavior can be attributed to the relatively low donor number of ether‐functionalized molecules compared to water, which limits their ability to solvate cations effectively [31].

In addition to solvation structure modulation, adsorption energy calculations were performed to evaluate the affinity of DMI molecules toward electrode surfaces. The adsorption energies of a water molecule and a DMI molecule on zinc and copper metal surfaces were calculated and compared (Figure 1g). On the Zn(002) surface, a water molecule exhibits an adsorption energy of −0.16 eV, whereas a DMI molecule adsorbs much more strongly with an adsorption energy of −1.50 eV, indicating that DMI adsorbs approximately nine times more strongly than water on the zinc metal surface. Similar trends were observed on the Cu(111) surface, where the adsorption energies were −0.22 and −1.73 eV for water and DMI, respectively, demonstrating that DMI adsorbs more than eight times more strongly than water. To experimentally verify the adsorption behavior, XPS analysis was conducted on zinc metal after immersion in each electrolyte for 24 h, followed by surface chemical species characterization (Figure S3). As a result, a distinct C─O bond signal was observed on the surface of zinc metal immersed in the DMI‐added electrolyte, whereas no corresponding C─O bond was detected on the zinc surface exposed to the blank electrolyte. This result experimentally confirms that DMI can adsorb onto the zinc metal surface. The adsorption of DMI is presumed to originate not only from the weak interactions associated with ether bonds, but also from non‐specific physical interactions. In particular, the bulky molecular framework of DMI may facilitate adsorption through van der Waals interactions and induced dipole moments as the molecule orients toward the zinc metal surface [32]. These results indicate that DMI molecules can preferentially adsorb onto both zinc and copper metal surfaces, enabling the formation of a DMI‐rich interfacial layer or an electrically structured interfacial double layer.

Through comprehensive electrolyte structural analysis combined with molecular dynamics simulations and adsorption energy calculations, it was confirmed that the addition of trace amount of DMI to aqueous zinc electrolytes can effectively modulate both the Zn^2+^ solvation structure and the hydrogen‐bonding network of water molecules. Unlike highly hydrophilic or strongly polar additives, DMI does not directly participate in the Zn^2+^ solvation shell; instead, it indirectly alters the solvation environment through interactions with water molecules. Moreover, adsorption energy calculations demonstrate the strong affinity of DMI toward zinc and copper metal surfaces, supporting the formation of a DMI‐rich adsorption layer or interfacial double layer. These combined effects, including altered solvation structures, modified water cluster configurations, and regulated electrode surface states are expected to facilitate uniform zinc deposition while suppressing interfacial corrosion in aqueous zinc metal batteries.

### 2.2 Effects of DMI Additive on Zinc Deposition/Stripping Performance

Following the theoretical insights into effect of DMI additive, we transitioned to empirical optimization to validate these findings in practical cell environments. To evaluate the applicability of DMI additives in aqueous batteries, coin cell tests were conducted using different concentrations. The optimization of additive concentrations was conducted prior to the electrochemical tests. The electrolytes containing 1–5 vol% of DMI were employed for the assessment. The prepared electrolytes were employed in the fabrication of Zn||Zn symmetric cells. Cycling tests conducted at a current density of 10 mA cm^−2^ for 0.2 h revealed that the electrolyte containing DMI 2 vol% exhibited the longest electrochemical operation, which outperforms among all candidates (Figure S4). Based on comprehensive results, the electrolyte containing 2 vol% of DMI was employed for all the following electrochemical tests.

**FIGURE 2:**
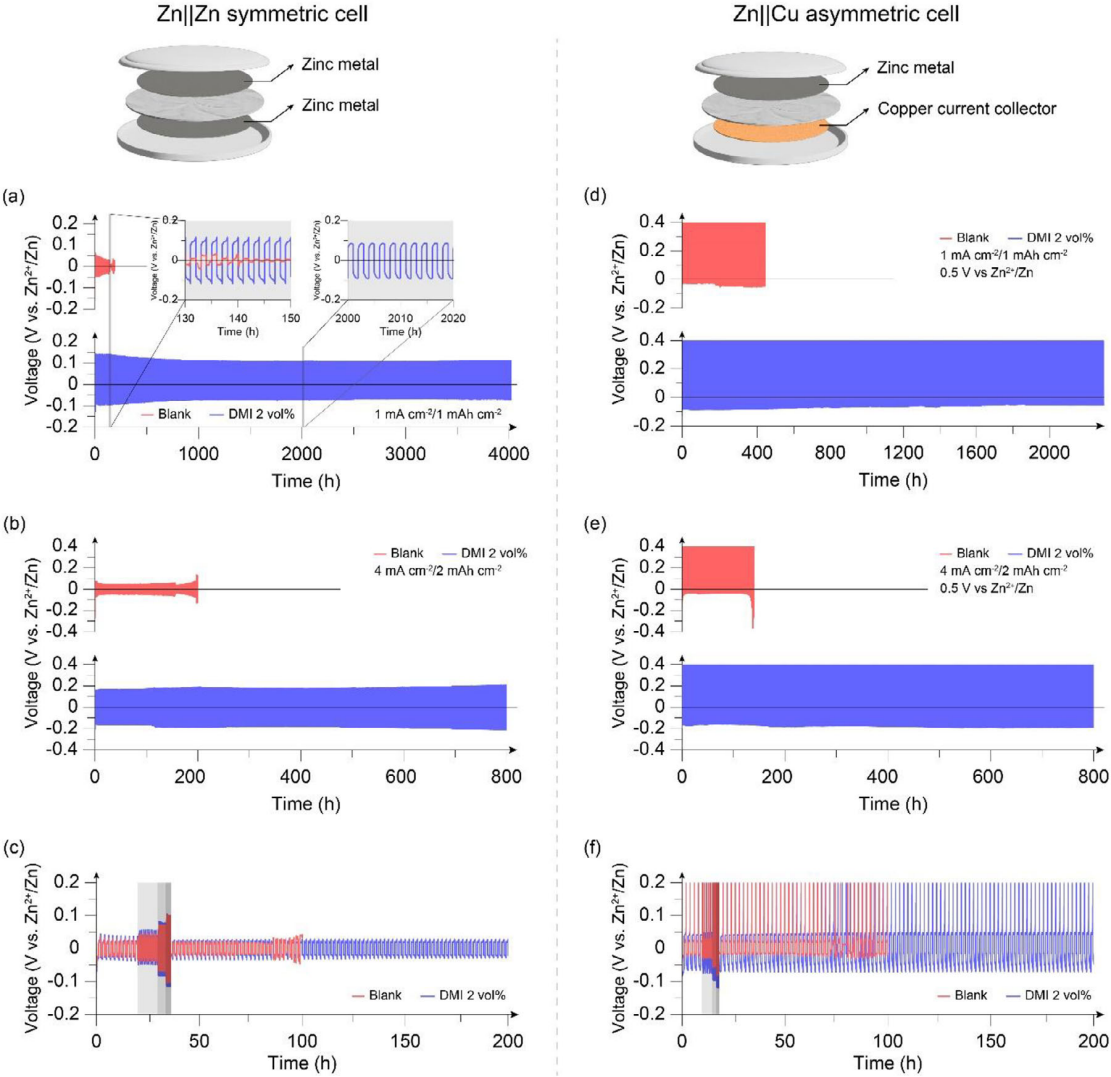
Cycling performance of Zn||Zn symmetric cells under (a) 1 mA cm^−2^/1 mAh cm^−2^ and (b) 4 mA cm^−2^/2 mAh cm^−2^ operation condition; (c) Rate capability of Zn||Zn symmetric cells from 1 to 10 mA cm^−2^ current density; Cycling performance of Zn||Cu asymmetric cells under (d) 1 mA cm^−2^/1 mAh cm^−2^ and (e) 4 mA cm^−2^/2 mAh cm^−2^ operation condition; (f) Rate capability of Zn||Cu asymmetric cells from 1 to 10 mA cm^−2^ current density.

To compare the difference in cell performance depending on the presence of DMI, a cycling test using Zn||Zn symmetric cells was first conducted. Figure 2a presents the cycling performance of symmetric cells evaluated under 1 mA cm^−2^/1 mAh cm^−2^ conditions. The cell using the blank electrolyte exhibited an internal short‐circuit after only 140 h of operation. In contrast to the cell with the blank electrolyte, the addition of DMI in the electrolyte enabled stable electrochemical operation for more than 4,000 h. Even at elevated current densities and capacities, DMI addition led to a substantial difference in electrochemical stability. As illustrated in Figure 2b, the cell with DMI‐added electrolyte exhibited a prolonged cycle life compared to the blank electrolyte cell, even under the harsh operating condition of 4 mA cm^−2^/2 mAh cm^−2^, without a significant increase in overpotential. This trend remained consistent during the rate capability tests conducted at a fixed capacity of 1 mAh cm^−2^ (Figure 2c). The results demonstrate that even after exposure to a high current density of 10 mA cm^−2^, the cell using DMI 2 vol% electrolyte exhibited prolonged cell stability compared to the control group. The initial deposition overpotential under 1 mA cm^−2^ of each cell was also discussed for evaluating zinc metal plating performance. (Figure S5) The comparison of the nucleation overpotentials reveals that the blank electrolyte exhibits a significantly higher value (137.0 mV) than the DMI‐added electrolyte (43.8 mV), indicating a larger resistance to initial zinc deposition during the first cycle in the blank electrolyte. This behavior is likely associated with the severe electrode corrosion occurring in the blank electrolyte at the early stage, resulting in a higher nucleation overpotential compared to the corrosion‐suppressed DMI‐added electrolyte (185.0 mV vs. 147.3 mV). In contrast, the zinc growth overpotential is higher in the DMI‐added electrolyte than in the blank electrolyte (103.5 mV vs. 48.0 mV). This increase can be attributed to the more uniform zinc growth induced by the DMI additive, which leads to a smaller increase in the effective surface area and consequently a higher actual current density compared to that of the blank electrolyte [33].

While Zn||Zn symmetric cell tests provide meaningful insights, results can be limited in evaluating the deposition/stripping efficiency due to the presence of an excessive zinc reservoir relative to the amount of zinc migrated during the cycles. To evaluate the cycling performance and coulombic efficiency simultaneously, asymmetric cell tests were conducted using alternative substrates. Among various substrates, copper foil was selected as the working electrode because it is widely used in zinc battery systems despite its well‐known tendency for non‐uniform zinc deposition [34, 35]. Interestingly, the DMI‐added electrolyte also achieved significant performance enhancements in the deposition/stripping processes on the copper current collector. As shown in Figure 2d, the addition of DMI to the electrolyte led to an approximately five‐fold increase in long‐term cycling performance under 1 mA cm^−2^/1 mAh cm^−2^ cycling conditions (70 h for blank electrolyte, over 2000 h for DMI 2 vol% added electrolyte). Furthermore, under high current density/high capacity and rate capability tests, which are similar to the symmetric cell evaluations, the Zn||Cu asymmetric cells using the DMI 2 vol% added electrolyte exhibited superior long‐term performance compared to the blank electrolyte cells (Figure 2e,f). Upon evaluating the deposition/stripping efficiency, the CE of the cells using the DMI‐added electrolyte approached 100% (Figures S6–S8). This indicates that the DMI additive effectively facilitates the long‐term reversibility of the zinc deposition/stripping process without inducing major side reactions.

### 2.3 Corrosion Inhibition Effect of DMI Additive

Based on the above cycling performance results, it is evident that the structural modulation of the electrolyte and the interfacial adsorption of DMI play a critical role in the observed performance enhancement of the DMI‐added electrolyte. Such improvement is primarily attributed to suppressed metal corrosion and more uniform zinc deposition induced by the altered electrolyte composition and modified surface state of the zinc electrode. To elucidate this mechanism, the corrosion‐inhibiting capability of the DMI electrolyte additive was systematically investigated.

**FIGURE 3:**
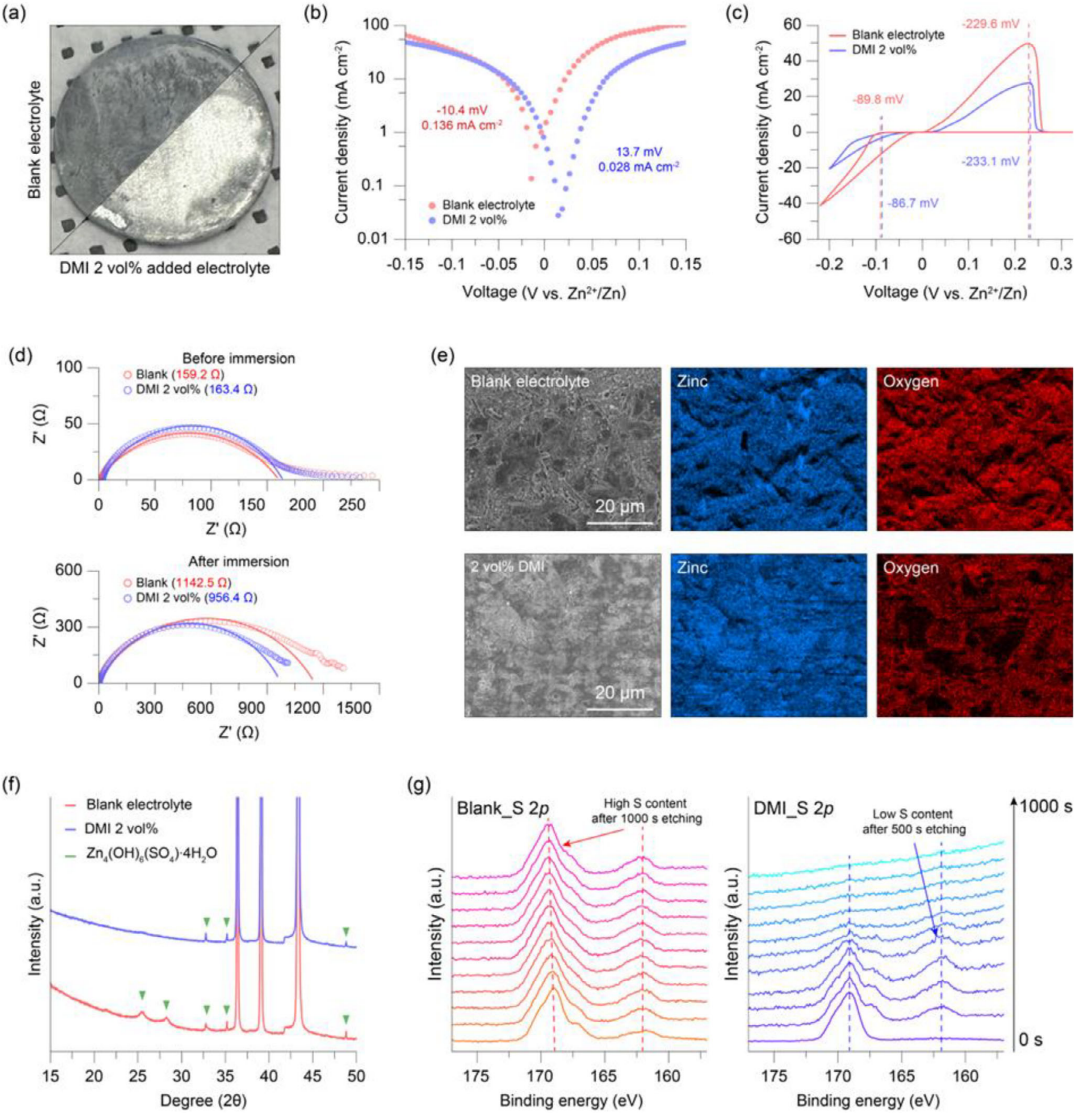
(a) Optical image of zinc electrodes after immersing in the blank electrolyte and DMI 2 vol% added electrolyte. (b) The Tafel plots of the Zn||Zn symmetric cell at the scan rate of 5 mV s^−1^. (c) Cyclic voltammogram (CV) of the Zn||Cu cells in different electrolytes. (d) Electrochemical impedance spectroscopy (EIS) curves of the Zn||Zn symmetric cells before/after immersing the zinc electrode in each electrolyte. (e) SEM images and EDS mapping results of the zinc electrode immersed for 14 days in the blank electrolyte and the DMI‐added electrolyte. (f) XRD patterns of zinc electrodes immersed in the blank electrolyte and the DMI‐added electrolyte. (g) XPS depth profile S 2*p* spectra of zinc electrodes immersed in the blank electrolyte and the DMI‐added electrolyte.

To qualitatively assess the corrosion inhibition effect of DMI, pieces of zinc anode were immersed in a blank electrolyte and DMI 2 vol% added electrolyte for two weeks, and the surface changes were visually examined (Figure 3a). As shown in the photographs, the zinc electrode stored in the DMI‐added electrolyte maintains its characteristic metallic feature. In contrast, the electrode immersed in the blank electrolyte exhibits surface by‐products resulting from side reactions. This phenomenon was equally evident on the copper current collectors; while the copper collector in the blank electrolyte turned completely black after storage, no visible corrosion was observed at current collectors stored in DMI‐added electrolyte (Figure S9). This indicates that DMI effectively suppresses metal corrosion in aqueous electrolytes even under static conditions. This corrosion suppression effect is indirectly associated with the electrolyte pH variation induced by the addition of DMI. Measurements of the actual pH values confirmed that both electrolytes fall within a similar acidic range of 3–5, suggesting that the difference in zinc dissolution arising solely from acidity is expected to be negligible (Figure S10). In addition, the lower pH of the DMI‐added electrolyte indicates a reduced hydroxide ion concentration, which suppresses the formation of ZBS, a representative corrosion byproduct formed on the metal anode surface. As a result, the decreased ZBS formation contributes to effective suppression of electrode corrosion.

To quantitatively evaluate the corrosion inhibition behavior of DMI, linear sweep voltammetry (LSV) measurements were conducted (Figure 3b). From the Tafel plot obtained in the blank electrolyte (red curve), the corrosion exchange current density and corrosion potential are determined to be 0.136 mA cm^−2^ and −10.4 mV vs. Zn^2+^/Zn, respectively. In comparison, the DMI‐added electrolyte exhibits a significantly lower corrosion exchange current density of 0.028 mA cm^−2^ and a more positive corrosion potential of 13.7 mV vs. Zn^2+^/Zn. These results clearly indicate that the presence of DMI effectively suppresses electrochemical corrosion at the metal surface [36]. Furthermore, cyclic voltammetry (CV) measurements reveal that the voltage hysteresis associated with zinc deposition/stripping is nearly identical regardless of DMI addition (Figure 3c). Low cathodic/anodic current densities are induced by DMI‐added electrolyte promoting uniform zinc deposition, resulting in a relatively small increase in the effective electrode surface area associated with dendritic zinc growth. Consequently, the peak currents of the zinc deposition/stripping peaks are lower. Moreover, the absence of a pronounced increase in peak potential with increasing DMI content suggests that the intrinsic zinc deposition/stripping overpotential is not significantly altered by the presence of DMI, in contrast to the behavior observed in the voltage profiles of zinc symmetric cells. To further examine the effect of DMI concentration, additional Zn||Cu half‐cell CV measurements were conducted using electrolytes with various DMI contents (Figure S11). The CV results reveal a clear difference between the blank electrolyte, DMI 1 vol% electrolyte and others, whereas the differences among the higher DMI concentrations are comparatively subtle. This behavior indicates that once the DMI concentration exceeds a certain threshold, changes induced during the initial CV cycles become less discernible. These results collectively demonstrate that the presence of DMI has a pronounced influence on the CV behavior and that a DMI content of 2 vol% represents an appropriate concentration for effective modulation of zinc deposition behavior. This observation indicates that DMI does not introduce additional kinetic barriers to zinc plating/stripping processes, nor does it induce undesirable side reactions, confirming its electrochemical compatibility with reversible zinc redox behavior. To further examine the impact of corrosion on interfacial electrochemical properties, Zn||Zn symmetric cells were assembled using electrodes immersed in each electrolyte for two weeks, and electrochemical impedance spectroscopy (EIS) measurements were performed before and after corrosion exposure. The Nyquist plots shown in the upper panel of Figure 3d correspond to electrodes prior to corrosion, while those in the lower panel represent electrodes after two weeks of immersion. The red curves correspond to the blank electrolyte, and the blue curves correspond to the DMI‐added electrolyte. Before corrosion, the charge transfer resistances are comparable for both electrolytes (159.2 and 163.4 Ω for the blank electrolyte and DMI‐added electrolyte, respectively), indicating similar initial interfacial kinetics. However, after two weeks of immersion, a pronounced increase in charge transfer resistance is observed for the blank electrolyte (1142.5 Ω), whereas the electrode exposed to the DMI‐added electrolyte exhibits a substantially lower resistance of 956.4 Ω. This reduced resistance confirms that DMI effectively mitigates corrosion‐induced interfacial degradation, thereby preserving favorable charge transfer characteristics.

To further examine corrosion‐induced interfacial degradation, the surface morphology of zinc electrodes immersed in each electrolyte for two weeks was investigated using scanning electron microscopy (SEM) (Figure 3e). The zinc metal surface exposed to the blank electrolyte exhibits the formation of thick and bulky corrosion byproducts, resulting in a highly rough and non‐uniform surface. In contrast, the zinc electrode immersed in the DMI‐added electrolyte shows significantly mitigated corrosion, maintaining a relatively smooth and uniform surface even after prolonged exposure. Elemental distribution analysis using energy‐dispersive spectroscopy (EDS) mapping further supports this observation. For the zinc surface corroded in the blank electrolyte, oxygen is detected uniformly across the entire electrode surface, indicating extensive oxidation and severe corrosion. In comparison, the zinc surface exposed to the DMI‐added electrolyte shows regions with markedly reduced oxygen signals, demonstrating that DMI effectively suppresses corrosion reactions on the zinc surface. X‐ray diffraction (XRD) analysis provides additional evidence for the corrosion‐inhibiting effect of DMI (Figure 3f). By comparing the XRD patterns of zinc electrodes corroded in each electrolyte, the electrode immersed in the blank electrolyte (red line) exhibits pronounced diffraction peaks corresponding to zinc basic sulfate, a typical corrosion byproduct formed in aqueous zinc electrolytes. In contrast, the zinc electrode corroded in the DMI‐added electrolyte (blue line) shows significantly weakened and fewer ZBS‐related peaks, indicating suppressed formation of corrosion products. These XRD results are consistent with the SEM and EDS observations and collectively confirm that DMI effectively mitigates zinc corrosion in aqueous electrolyte environments. For deeper insight into the corrosion layers, XPS depth profiling was performed while etching the electrode surface with a 1 keV Ar^+^ ion beam (Figure 3g). In the electrode corroded in the blank electrolyte, the S 2*p* spectra revealed a clear SO_4_^2−^ signal at 169.8 eV on the surface, indicating the presence of sulfate anion‐containing corrosion products. Even after more than 1000 s of Ar^+^ etching, the SO_4_^2−^ signal was still detected in the S 2*p* spectra, demonstrating that the corrosion layer extended deeply beneath the zinc surface. In the electrode corroded in the DMI‐added electrolyte, the SO_4_^2−^ signal was also detected on the outermost surface, consistent with the increased resistance observed in EIS and the partial oxidation seen in SEM and XRD. However, after only 400 s of Ar^+^ etching, the SO_4_^2−^ signal nearly disappeared, indicating that the corrosion layer formed in the DMI‐added electrolyte was significantly thinner than that in the blank electrolyte. The corrosion layer thickness was estimated from the known Ar^+^ beam energy and etching rate for metal surfaces. The blank electrolyte produced a layer thicker than 0.83 µm, whereas the DMI‐added electrolyte resulted in a substantially thinner layer of approximately 0.34 µm, clearly demonstrating that DMI effectively suppresses corrosion on the zinc electrode surface. Furthermore, consistent results were obtained from the depth‐dependent comparison of O 1*s* spectra using XPS depth profiling (Figure S12). For the zinc metal corroded in the blank electrolyte, the corrosion layer remains intact throughout the sputtering process, showing negligible changes in peak intensity even after 1000 s, which is consistent with the behavior observed in the S 2*p* spectra. This indicates the formation of a thick and persistent corrosion layer on the zinc surface. In contrast, for the zinc metal corroded in the DMI‐added electrolyte, the intensities of the peaks corresponding to zinc oxide and ZBS decrease sharply after approximately 600 s of sputtering. This rapid attenuation of corrosion‐related signals suggests the formation of a significantly thinner and less stable corrosion layer. These results provide clear and direct evidence that the DMI additive effectively suppresses zinc corrosion in aqueous electrolytes.

To elucidate how corrosion triggered by the electrolyte affects the overall electrochemical stability, shelving recovery tests were conducted. In both symmetric and asymmetric cell configurations, cells using the DMI‐added electrolyte demonstrated stable long‐term performance (Figure S13). These results support the corrosion‐inhibiting effect of DMI substantially facilitating stable electrochemical operation.

### 2.4 Uniform Zinc Deposition Triggered by DMI Additive

Overall, the preceding results demonstrate that DMI effectively suppresses zinc corrosion, reduces ZBS formation, and improves the reversibility of zinc metal cycling. These findings indicate that DMI is a promising electrolyte additive for enhancing the interfacial stability of zinc metal anodes in aqueous battery systems.

To evaluate the effectiveness of DMI as an electrolyte additive, zinc deposition and stripping behaviors were systematically examined using a Zn||Zn symmetric cell equipped with *operando* top‐view optical visualization. The cell was operated at a current density of 1 mA cm^−2^ with an areal capacity of 1 mAh cm^−2^, followed by a stripping step. In the conventional electrolyte, zinc deposition is highly nonuniform, with dark dendritic features forming preferentially at localized regions on the anode surface (Figure S14 and Video S1). As shown in image (iv), the fully plated electrode exhibits isolated yet pronounced black dendritic zinc clusters, indicating intrinsically uneven zinc nucleation and growth in the absence of additives. Notably, even after the stripping process is completed (image (vi)), residual dendritic structures remain attached to the electrode surface. The persistence of these remnants confirms the formation of dead zinc, a well‐established degradation pathway that causes irreversible active material loss and gradual failure of aqueous zinc batteries. In contrast, *operando* imaging of the cell employing the DMI‐added electrolyte shows a significantly improved deposition behavior (Figure S15 and Video S2). With the introduction of DMI, zinc nucleation and growth become markedly more uniform over the entire electrode surface. As illustrated in image (iv), dendritic growth is effectively suppressed during deposition, and the electrode displays a homogeneous color change, indicative of even zinc plating without the formation of protrusions. This behavior implies that DMI establishes a more controlled interfacial environment, likely by regulating the interfacial solvation structure and stabilizing the electric double layer. Moreover, after complete stripping (image (vi)), the electrode surface remains largely clean, with no discernible dead zinc residues. The near‐complete reversibility of zinc deposition and stripping under DMI‐added conditions demonstrates that the additive promotes stable plating/stripping processes and effectively suppresses irreversible zinc accumulation.

**FIGURE 4:**
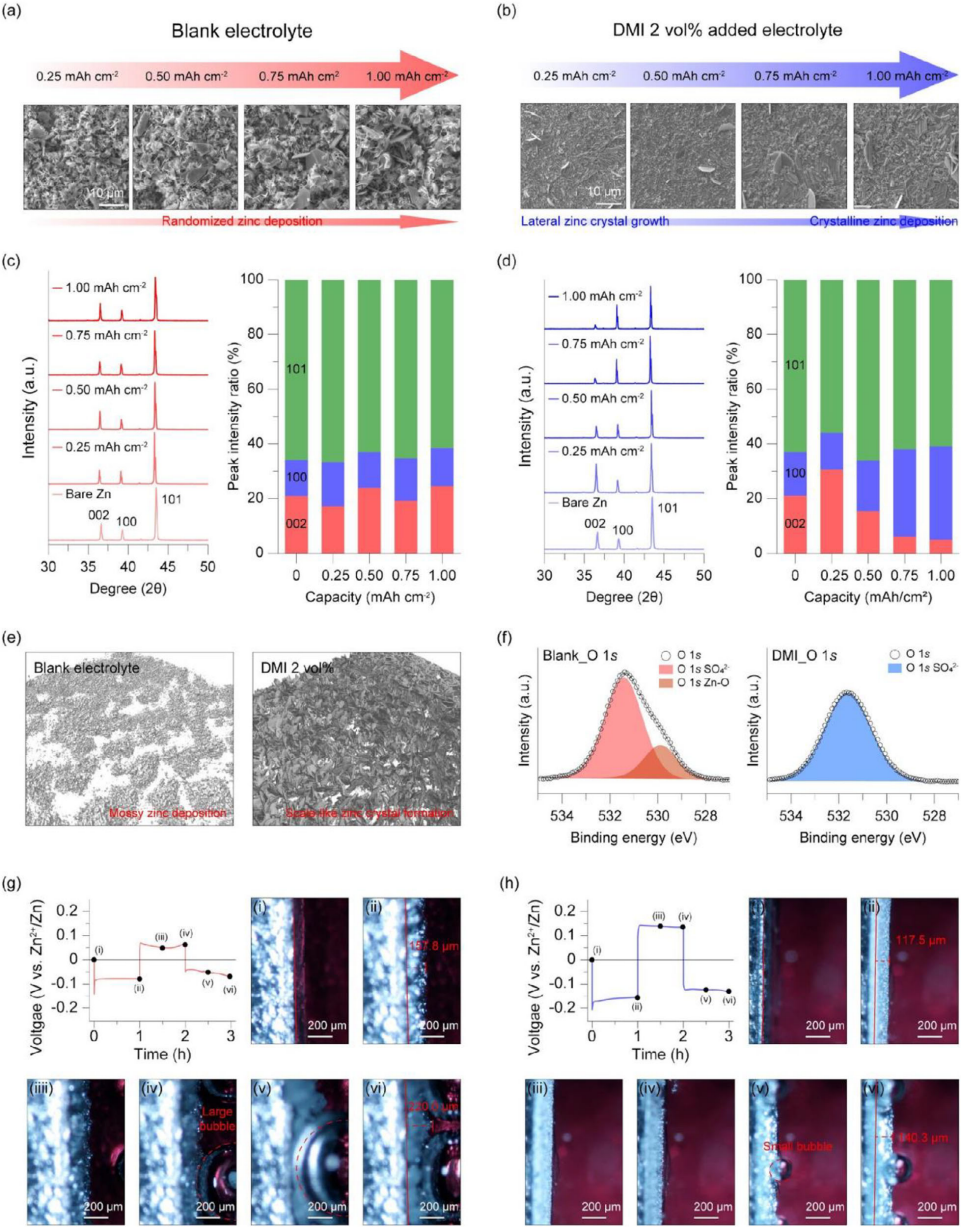
SEM images of zinc electrode after zinc deposition (a) in the blank electrolyte, (b) DMI‐added electrolyte at 1 mA cm^−2^ for different capacities. XRD patterns and peak intensity ratios of zinc electrode after zinc deposition in (c) the blank electrolyte, (d) DMI‐added electrolyte at 1 mA cm^−2^ for different capacities. (e) 3D CT images of the zinc electrode after zinc deposition in the blank electrolyte and the DMI‐added electrolyte. (f) XPS O 1*s* spectra of the zinc electrode in different electrolytes after zinc deposition. (g) Cross‐section *operando* optical images and voltage profile of the Zn||Zn symmetric cell at 40 mA cm^−2^ and 40 mAh cm^−2^: (g) the blank electrolyte and (h) the DMI‐added electrolyte. Optical images of (i)–(vi) indicate (i) pristine, (ii) 1 h deposition, (iii) 30 min stripping, (iv) 1 h stripping, (v) 30 min re‐deposition, and (iv) 1 h re‐deposition, respectively.

To confirm that the uniform zinc growth suggested by *operando* top‐view imaging corresponds to the actual deposition morphology, SEM characterization was performed on electrode surfaces after zinc deposition at different areal capacities. Zinc was deposited onto pristine zinc electrodes at a current density of 1 mA cm^−2^ with areal capacities of 0.25, 0.5, 0.75, and 1.00 mAh cm^−2^, and the resulting surface morphologies were examined. In the blank electrolyte, zinc deposited at all capacities exhibited irregular, non‐directional growth without any clear morphological trend (Figure 4a). By contrast, in the DMI‐added electrolyte, SEM images show that zinc initially grows perpendicular to the electrode surface up to a capacity of 0.25 mAh cm^−2^ (Figure 4b). With increasing deposited capacity, zinc grains gradually become more pronounced, evolving into a granular morphology. Although not distinguished by the naked eye, this granular zinc structure alters the light absorption of the electrode surface, leading to a reduction in overall brightness. This effect accounts for the gradual color change observed in the *operando* top‐view imaging.

The capacity‐dependent evolution of zinc growth orientation under DMI‐added electrolyte conditions was further corroborated by XRD analysis. Diffraction peaks at 36.4°, 39.1°, and 43.3° can be indexed to the (002), (100), (101), (103), and (110) planes of hexagonal zinc. In the blank electrolyte, no preferred crystallographic orientation was observed at any deposition capacity, indicating random zinc growth (Figure 4c). Such random deposition under 1 M ZnSO_4_ conditions is conducive to dendritic zinc formation. In contrast, upon the addition of 2 vol% of DMI, the relative intensity of the Zn(002) reflection increased from 21.1% to 30.6% during the initial deposition stage (∼0.2 mAh cm^−2^), suggesting preferential growth along the (002) plane (Figure 4d). At higher capacities, however, the contribution of the Zn(002) plane decreased substantially from 15.4% to 4.9%. Meanwhile, the fraction of the Zn(100) plane initially declined from 15.9% to 13.6% but then rose sharply to 34.2% at a deposition capacity of 1.0 mAh cm^−2^. This shift in zinc growth behavior can be attributed to the presence of DMI, which likely promotes initial zinc deposition along the (002) direction through the formation of a molecular adsorption layer on the electrode surface. As deposition progresses, alterations in the surface chemistry, potentially arising from DMI decomposition and the development of an additional interfacial layer, may redirect zinc growth toward the (100) plane. Given that such dynamic, capacity‐dependent changes in zinc crystallographic orientation have rarely been reported, further studies are needed to clarify the underlying mechanisms in detail.

To obtain a more rigorous assessment, additional morphology and chemical analysis were introduced. 3D computed tomography (CT) analysis was conducted on the zinc metal anode to visualize a 3D image of the zinc surface after deposition (Figure 4e). A 3D image of zinc deposited in blank electrolyte demonstrates mossy zinc dendrites, indicating uneven, randomized zinc deposition. In contrast, the zinc surface after zinc deposition in DMI 2 vol% added electrolyte displays crystalline zinc growth, consistent with SEM and XRD analysis results. XPS analysis of the anode surface after zinc deposition also demonstrated that DMI‐added electrolyte mitigates zinc corrosion during the zinc deposition process (Figure 4f). O 1*s* spectrum of zinc metal after deposition in blank electrolyte, zinc oxide and ZBS peak are shown, indicating the occurrence of severe zinc corrosion along with zinc deposition reaction. However, the O 1*s* spectrum of zinc metal after deposition in DMI‐added electrolyte demonstrates low ZBS peak intensity and no zinc oxide peak, implying the reduction of zinc corrosion in DMI‐added electrolyte. This corrosion inhibition effect of DMI additive during zinc deposition reaction is consistent with the zinc corrosion analysis during zinc immersion in DMI‐added electrolyte as described in the previous chapter.

Furthermore, *operando* side‐view visualization was carried out under high current density and high areal capacity conditions, where interfacial instability is more severe. The Zn||Zn symmetric side‐view optical cell was cycled at a current density of 40 mA cm^−2^ with a capacity of 40 mAh cm^−2^. Figure 4g and Video S3 show the operando side‐view images obtained from a cell using 1 m ZnSO_4_ electrolyte. As seen in images (i) and (ii), the electrode surface becomes progressively rough during zinc deposition, with pronounced surface irregularities and localized protrusions forming. During the subsequent stripping process (images (iii) and (iv)), zinc removal occurs in a highly uneven manner, resulting in an irregularly eroded surface accompanied by substantial residues of dark dead zinc. Notably, image (v) reveals the formation of a large gas bubble originating from hydrogen evolution, evidencing the occurrence of parasitic side reactions under these harsh cycling conditions. In the following deposition step, this bubble hinders the ionic transport and locally suppresses zinc deposition, thereby further exacerbating dendritic growth, as shown in images (vi) and (vii). Overall, these side‐view observations confirm that severe dendrite formation and intense hydrogen evolution take place in the blank electrolyte under high‐current, high‐capacity operation. In contrast, when the DMI‐added electrolyte is employed, zinc deposition and stripping occur in a much more uniform and stable manner (Figure 4h; Video S4). As shown in images (i) and (ii), the electrode surface remains comparatively smooth throughout deposition, unlike the significant surface roughening observed in the blank electrolyte. This behavior clearly indicates more homogeneous zinc growth in the presence of DMI. Quantitative comparison of the deposited zinc layer further corroborates this result: the blank electrolyte forms a porous zinc layer with a thickness of 157.8 µm, whereas the DMI‐added electrolyte yields a denser zinc layer with a reduced thickness of 111.75 µm, implying fewer internal voids and a more compact structure. During the subsequent stripping process (images (iii) and (iv)), zinc is removed uniformly across the electrode surface, which remains smooth with minimal dead zinc formation. The following deposition cycle (images (v) and (vi)) again demonstrates even zinc plating over the entire surface. In addition, significantly fewer hydrogen bubbles are observed during deposition and stripping in the presence of DMI, indicating effective suppression of parasitic reactions and mitigation of zinc corrosion. Such enhanced interfacial stability is crucial for achieving uniform zinc morphology and improving the reversibility of the zinc metal anode.

### 2.5 Electrochemical Performance of Full Cell Using DMI Additive

**FIGURE 5:**
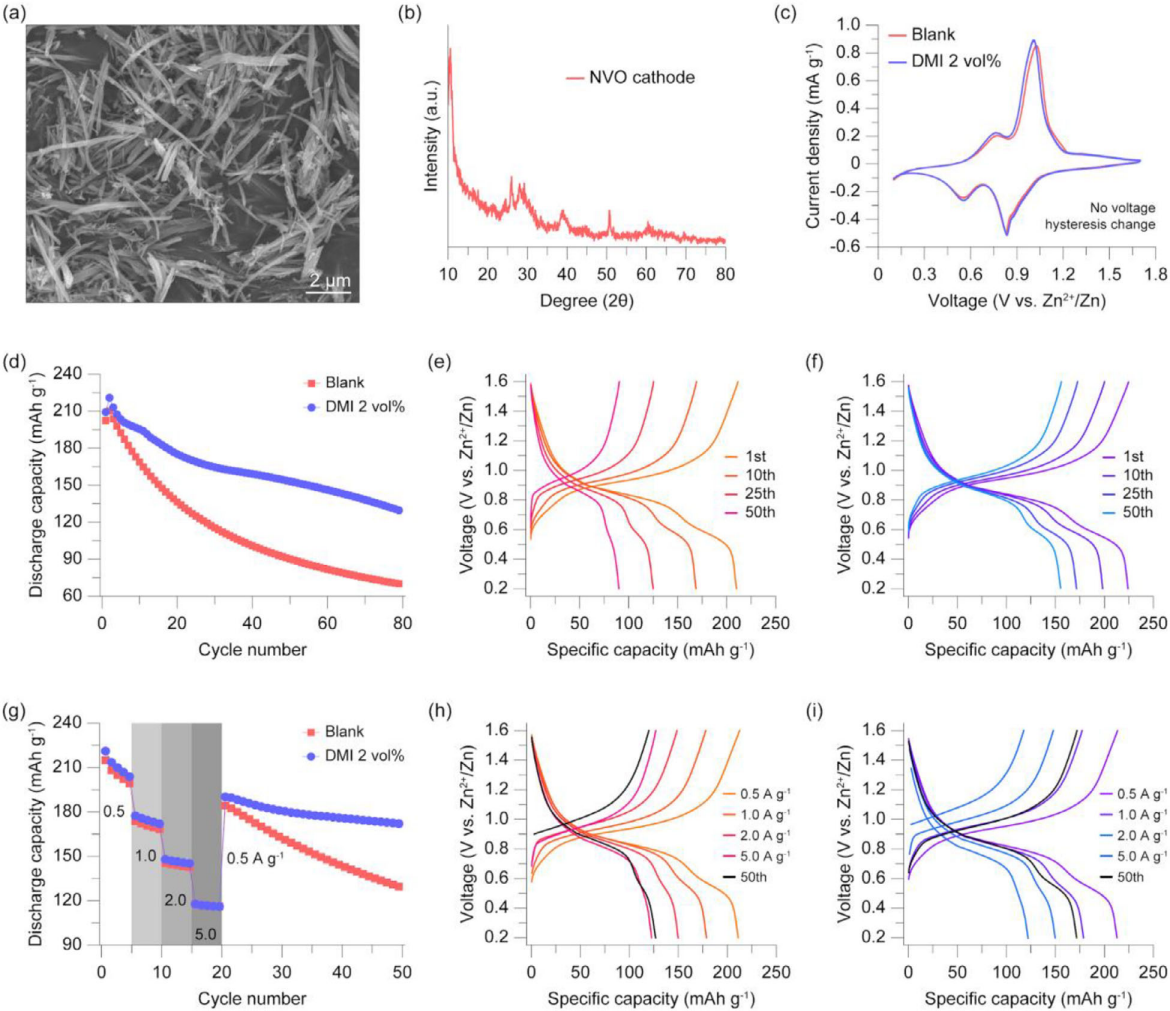
NVO full cell cycling data. (a) SEM image of synthesized NVO particles. (b) XRD pattern of synthesized NVO particles. (c) CV plots of the NVO||Zn cells using different electrolytes. (d) Cycling stability test of NVO||Zn cells using different electrolytes operated under 0.2 A g^−1^ current density. Voltage profiles of NVO||Zn cell using (e) blank electrolyte and (f) DMI 2 vol% electrolyte, respectively. g) Rate capability of NVO||Zn cells using different electrolytes. h,i) The corresponding voltage profiles for Figure 5g; (h) NVO||Zn cell using blank electrolyte. (i) NVO||Zn cells using DMI 2 vol% electrolyte.

To assess the applicability of the DMI‐added electrolyte in practical systems, full cells employing sodium vanadate (NVO) cathodes were fabricated. The NVO active material was synthesized according to previous reports [23, 37, 38]. SEM image confirmed that the synthesized material exhibited the identical morphology and the crystal structure characterized by XRD analysis also confirmed that the material was successfully prepared (Figure 5a,b). First, CV tests were performed to investigate the changes in electrochemical behavior depending on the presence of the DMI additive. Under the same conditions, the cell using the DMI‐added electrolyte exhibited a CV profile identical to the control group (Figure 5c). This indicates that the DMI additive does not alter the intrinsic electrochemical reactions of the NVO material. Next, long‐term stability tests were conducted to assess the durability of the NVO||Zn full cells. Figure 5d shows the capacity profiles of cells operated under a current density of 0.2 A g^−1^. After 80 cycles, the cell using the 2 vol% DMI electrolyte maintained 63.3% of its initial capacity, which is superior to the blank electrolyte cell that retained only 49.1%. Also, by comparing the discharge capacities at the same cycle number, the cell with the DMI‐added electrolyte delivered higher capacity than the control. This result is attributed to the corrosion inhibition and uniform zinc deposition facilitated by the DMI additive. Additionally, the observed extra capacity is closely related to the reaction mechanism of the NVO cathode. Unlike cathodes that store charge via simple Zn^2+^ intercalation, NVO is known to exhibit capacity through a Zn^2+^/H^+^ co‐intercalation mechanism. In this regard, the relatively lower pH of the DMI‐added electrolyte is expected to promote H^+^ intercalation, leading to enhanced capacity [38]. Moreover, capacity fading in NVO cathodes has been widely attributed to vanadium ion dissolution, typically in the form of VO_2_^+^, followed by parasitic reactions at both the cathode and anode that result in the formation of electrochemically inactive vanadium compounds, such as Zn_3_(OH)_2_V_2_O_7_·2H_2_O [39]. The lower pH environment induced by the DMI‐added electrolyte is therefore anticipated to suppress these side reactions, thereby mitigating capacity degradation. As expected, in the rate capability evaluations, the DMI 2 vol% cell significantly outperformed the blank electrolyte. As confirmed in Figure 5g, the DMI‐added cell exhibited no significant capacity decay even when operated at higher scan rates, indicating the absence of additional degradation caused by the additive. Upon returning to the initial rate, the cell retained 80.6% of its original capacity, a value significantly higher than that of the blank electrolyte cell, which experienced extensive capacity fading (Figure 5h,j). Even at a high current density of 2.0 A g^−1^, the cell with 2 vol% DMI exhibited superior capacity retention compared to the blank electrolyte cell (Figure S16). This indicates that the uniform zinc deposition induced by DMI remains effective under high current conditions, signifying the development of a highly adaptable aqueous zinc battery system suitable for a wide range of practical applications.

## 3 Conclusion

In this work, DMI was demonstrated as an effective electrolyte additive for stabilizing zinc metal anodes in aqueous zinc batteries. Through a combination of spectroscopic analysis, molecular simulations, and electrochemical characterization, it was revealed that DMI indirectly modulates the Zn^2+^ solvation structure by restructuring the hydrogen‐bond network of water rather than directly coordinating with Zn^2^^+^. This weakened solvation environment facilitates Zn^2+^ desolvation while suppressing water‐induced parasitic reactions. In parallel, adsorption energy calculations and experimental results confirmed the strong affinity of DMI toward metallic zinc, enabling the formation of a molecular adsorption layer that effectively regulates the electrode–electrolyte interface. Thanks to these synergistic effects, the DMI‐added electrolyte significantly suppresses zinc corrosion, hydrogen evolution, and ZBS formation. Furthermore, operando optical visualization combined with SEM, XRD, and 3D CT analyses revealed that DMI not only promotes uniform and compact zinc deposition but also induces a capacity‐dependent evolution in zinc crystallographic growth behavior. Specifically, DMI enables regulated zinc growth at the initial deposition stage and maintains stable, dense deposition even at higher areal capacities, effectively inhibiting dendrite formation and dead zinc accumulation throughout extended cycling. Overall, this study establishes a comprehensive mechanistic understanding of how a weakly polar, ether‐based additive can simultaneously regulate electrolyte structure and interfacial chemistry while dynamically controlling zinc growth behavior as a function of deposition capacity, thereby overcoming the coupled degradation pathways of aqueous zinc metal anodes. The demonstrated strategy provides a simple, cost‐effective, and scalable electrolyte engineering approach, offering a promising route toward high‐performance, long‐life, and safe aqueous zinc batteries for large‐scale energy storage applications.

## 4 Experimental Section

### 4.1 Electrolyte Synthesis

Solvent for DMI‐added electrolyte was obtained by mixing *x* mL of DMI and (100‐*x*) mL of deionized water (*x* = 0–5). After making the composite solvent, the DMI‐added electrolyte was prepared by dissolving 0.1 mol of ZnSO_4_·7H_2_O in the composite solvent to a total volume of 100 mL.

### 4.2 Sodium Vanadate (NVO) Cathode Preparation

5.0 g of V_2_O_5_ powder was mixed with 50 mL of 2 m NaCl aqueous solution. The mixed solution was stirred at 25°C for 3 days, resulting in a change in solution color from orange to reddish orange. Following the reaction, the mixed solution was centrifuged and washed with deionized water and ethanol three times, then dried at 70°C for 24 h. Finally, dark red NVO nanofiber powder was obtained.

### 4.3 Electrolyte Characterization

The fundamental structures of the electrolytes were characterized using Fourier‐transform infrared spectroscopy (FTIR; Thermo Fisher Scientific Nicolet IS50) and Raman spectroscopy (Thermo Fisher DXR3xi). Raman spectra were recorded with a 532 nm excitation laser. Contact angle measurements with different electrolytes were performed using a contact angle meter (Kruss DSA 25). The crystal structures of all zinc electrodes were analyzed by X‐ray diffraction (XRD; Rigaku Smartlab) with Cu Kα radiation (λ = 1.5406 Å) over a 2θ range of 10°–80° and a step size of 0.02°. X‐ray photoelectron spectroscopy (XPS) spectra were collected using a Sigma Probe (Thermo Fisher Scientific, UK) with a monochromated Al Kα source (1486.6 eV). CasaXPS software was employed to analyze the spectra. Top‐view and cross‐sectional scanning images, along with EDS elemental maps, were obtained using field emission scanning electron microscopy (FE‐SEM; Hitachi, Regulus 8230) at 10 kV.

### 4.4 Electrochemical Measurements

CR2032 coin cells were employed for all electrochemical measurements in this study, and the tests were carried out on a WonATech cycler at 25°C. Porous glass fiber (Whatman, GF/C) was used as the separator, and the electrolyte volume in each cell was 100 µL. For Zn||Zn symmetric cells, two zinc foils punched in 16 mm diameter discs were used. Zn||Cu half‐cells were assembled using copper foil as the cathode and zinc foil as the anode. The NVO||Zn full cells were constructed with zinc foils (16 mm diameter) and NVO electrodes (11 mm diameter). For Tafel plot measurements, 16 mm diameter zinc disks were used as both the working and counter electrodes. Cyclic voltammetry (CV) was conducted with a Zn||Cu cell by scanning from −0.2 to 0.5 V at 0.1 mV s^−1^. Electrochemical impedance spectroscopy (EIS) of Zn||Zn symmetric cells was performed with a 10 mV amplitude over the frequency range of 1 MHz–0.1 Hz.

The cycling stability of Zn||Zn symmetric cells and Zn||Cu half‐cells was tested at 1 mA cm^−2^ with a capacity of 1 mAh cm^−2^, while the rate performance was examined at a fixed areal capacity of 1 mAh cm^−2^ under current densities of 1, 2, 5, and 10 mA cm^−2^ for 10 cycles, followed by recovery cycling at 1 mA cm^−2^, 1 mAh cm^−2^. The high current/high‐capacity test was conducted at 1 mA cm^−2^ with a capacity of 1 mAh cm^−2^. The stripping cut‐off voltage of Zn||Cu half‐cells was 0.5 V versus Zn^2+^/Zn. For shelving recovery tests, assembled coin cells were cycled under 1 mA cm^−2^, 1 mA h cm^−2^ conditions for *x* cycles followed by *x* h rest (*x* = 10 for Zn||Cu cell and 20 for Zn||Zn cell). The cycling conditions were repeated until meaningful results were obtained. For full cell tests, NVO served as the cathode and zinc electrode as the anode. Galvanostatic charge/discharge tests of full cells were conducted at 0.2 and 2.0 A g^−1^ within a voltage range of 0.2–1.6 V versus Zn^2+^/Zn. Cyclic voltammetry (CV) of NVO||Zn full cells was performed by scanning between 0.2 and 1.6 V at a scan rate of 0.2 mV s^−1^.

### 4.5 *Operando* Optical Visualization

The cells for *operando* optical microscopy were constructed using a commercially available handmade cell. All optical cells were prepared under the same conditions as the original two‐electrode cells corresponding to each battery system. *Operando* visualization was recorded using optical microscopy (50× lens, Nikon LV150N). The electrode was observed through a 3 mm diameter aperture in the custom‐made cell structure, which was sealed with a 10 mm diameter glass window.

### 4.6 Molecular Dynamics Simulations

Molecular dynamics (MD) simulations were performed using LAMMPS (Ver. 23 Jun 2022) [40]. The solvent force field parameters were generated by the LigParGen2.1 using the OPLS‐AA force field due to that it is widely applied in organic liquids and electrolyte simulations [41, 42]. Besides, advanced restrained electrostatic potential (RESP2) atomic partial charges were adopted, which were derived from the electrostatic potential (ESP) charges using the Multiwfn program (Ver. 3.8) [43]. The initial atomic coordinates were generated by PACKMOL (Ver. 20.3.5), and the MD snapshots were visualized by VESTA (Ver. 4.6.0) [44, 45]. The time step was fixed to be 1 fs for all simulations. All systems were first equilibrated in the isothermal‐isobaric (constant NPT) ensemble using the Parrinello‐Rahman barostat for 5 ns to maintain a temperature of 298 K and a pressure of 1 atm with time constants of 0.1 and 1 ps, respectively [46]. Subsequently, the systems were heated from 298 to 340 K within 5 ns and maintained at 340 K for 5 ns, followed by annealing from 340 to 298 K within 5 ns. After that, all systems were equilibrated at 298 K in a constant NPT ensemble for another 5 ns. A production run of 12 ns at 298 K in the canonical (constant NVT) ensemble under Nosé–Hoover thermostat was finally conducted, which was used to analyze. The last 5‐ns NVT simulations were output every 1000 steps (i.e., a total of 5000 frames) and used for the analysis of solvation structures.

### 4.7 Density Functional Theory Calculations

The periodic density functional theory calculation was conducted in Vienna Ab initio Simulation Package (VASP, 6.4.3) [47] and the results were visualized in the VESTA (Ver. 4.6.0). The Perdew–Burk–Ernzerhof generalized gradient approximation functional was adopted. The energy cutoff was set to 520 eV. The self‐consistent field and geometry convergence tolerance were set to 1 × 10^−5^ and 1 × 10^−4^ eV, respectively. The adsorption energy was defined as follows:

$$\;E_{ads}=E_{surface-molecule}\;-E_{surface}-E_{molecule}$$

where *E*_surface–molecule_, *E*_surface_, and *E*_molecule_ are the total energy of the surface–molecule system, the clean surface, and the isolated molecule, respectively.

## Conflicts of Interest

The authors declare no conflicts of interest.

## Supporting information




**Supporting File 1**: smtd70591‐sup‐0001‐SuppMat.docx.

**Supplemental File 2**: smtd70591‐sup‐0002‐VideoS1.mp4.

**Supporting File 3**: smtd70591‐sup‐0003‐VideoS2.mp4.

**Supporting File 4**: smtd70591‐sup‐0004‐VideoS3.mp4.

**Supporting File 5**: smtd70591‐sup‐0005‐VideoS4.mp4.

## Data Availability

The data that support the findings of this study are available from the corresponding author upon reasonable request.
