# Supplementary material for: Weakly Polar Organic Additive Inducing Capacity‐Dependent Zinc Growth Transition via Indirect Solvation and Adsorption Engineering in Aqueous Electrolytes
Source: Small Methods. 2026 Mar 19;10(8):e02427. doi: 10.1002/smtd.202502427 (PMC13103614; doi:10.1002/smtd.202502427)
Supplement: Supplementary file 1 — Supporting File 1: smtd70591‐sup‐0001‐SuppMat.docx. [file SMTD-10-e02427-s004.docx]

Supporting Information

**Weakly Polar Organic Additive Inducing Capacity-Dependent Zinc Growth Transition via Indirect Solvation and Adsorption Engineering in Aqueous Electrolytes**

*Sung-Ho Huh^a†^, Beom-Keun Cho^a†^, Yao-Peng Chen^b,c^, So Hee Kim^d^ Jong-Seong Bae^e^, Xiang Chen^b,c*^ and Seung-Ho Yu^a,f*^*

S.-H. Huh, B.-K. Cho, Prof. S.-H. Yu

^a^ *Department of Chemical and Biological Engineering, Korea University, Seoul, Republic of Korea*

E-mail: seunghoyu@korea.ac.kr (Seung-Ho Yu)

Y.-P. Chen, X. Chen

*^b^ Beijing Key Laboratory of Complex Solid State Batteries & Tsinghua*

*Center for Green Chemical Engineering Electrification, Department of Chemical Engineering, Tsinghua University, Beijing 100084, P. R. China*

*^c^ The Innovation Center for Smart Solid State Batteries, Yibin 644002, P. R. China*

E-mail: xiangchen@mail.tsinghua.edu.cn (Xiang Chen)

S. H. Kim
*^d^ Advanced Analysis Center, Korea Institute of Science and Technology (KIST), Seoul 02792, Republic of Korea*

J.-S. Bae *^e^* *Busan Center, Korea Basic Science Institute (KBSI), 30 Gwahaksandan 1-ro, Gangseo-gu, Busan, 46742, Republic of Korea*

S.-H. Yu

*^f^* *Department of Battery-Smart Factory, Korea University, Seoul, Republic of Korea*

E-mail: seunghoyu@korea.ac.kr (Seung-Ho Yu)

Funding: Korea Institute of Marine Science & Technology Promotion (KIMST) (RS-2025-16322969), National Research Foundation of Korea (NRF) (No. RS-2025-25441256), Beijing Municipal Natural Science Foundation (L247015 and L233004), The Ministry of Science and ICT in Korea via KBSI (Grant No. C524100)

Keywords: Aqueous electrolytes, Zinc metal anodes, Electrolyte additives, Corrosion inhibition, Zinc deposition

^†^ Sung-Ho Huh and Beom-Keun Cho contributed equally to this work

^*^ Corresponding author


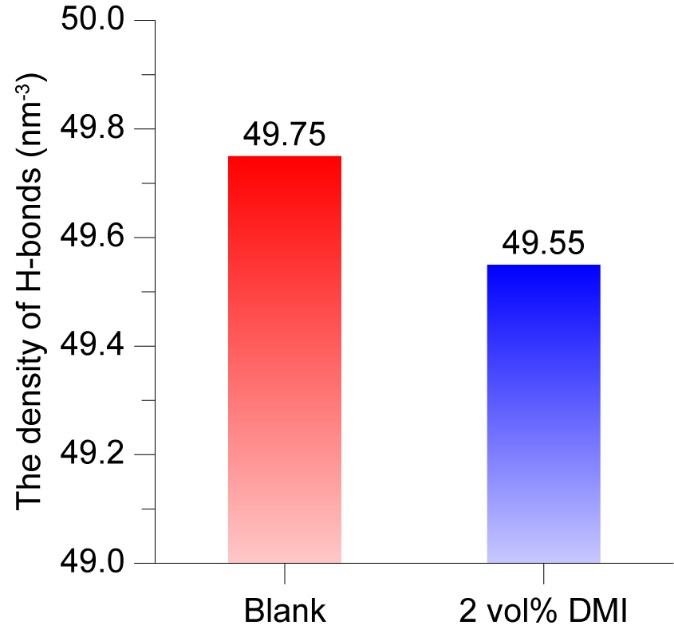


**Figure S1**. Number of hydrogen bonds per unit volume with and without DMI.


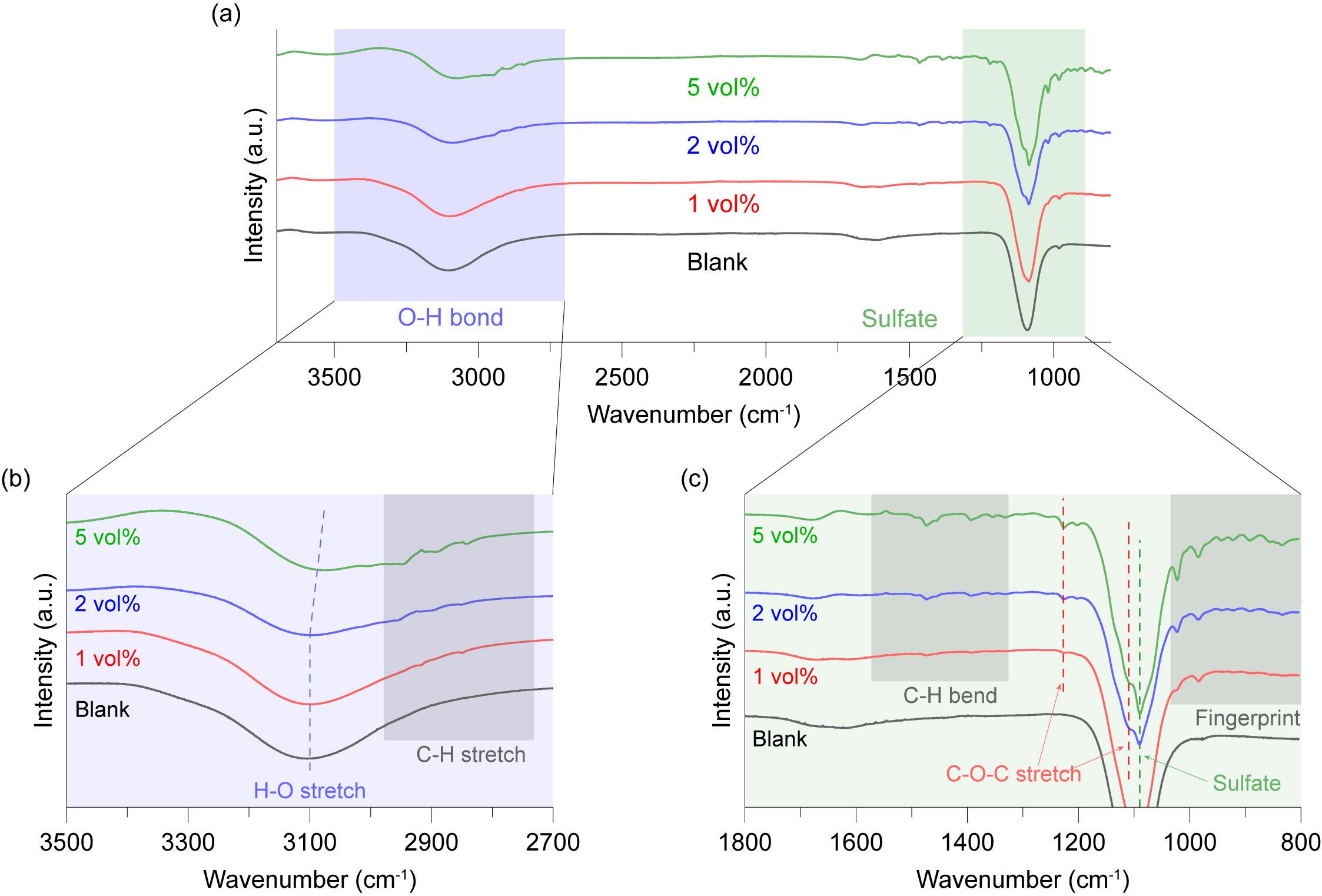


**Figure S2.** a) FTIR spectra of the blank electrolyte and the DMI-added electrolytes. b) Magnified XRD pattern representing b) in the 3500–2700 cm^–1^ range and ii) in the 1800–800 cm^–1^ range.


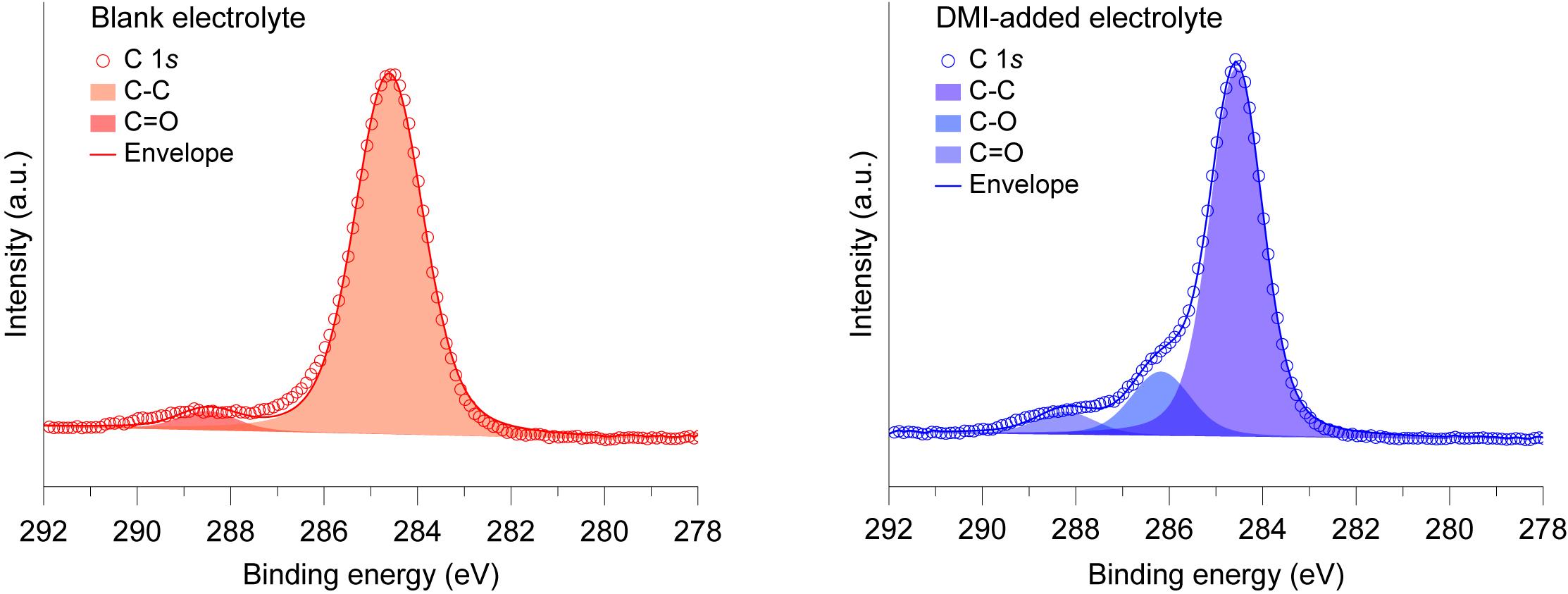


**Figure S3.** XPS C 1*s* spectra of the zinc electrode immersed in the blank electrolyte and the DMI-added electrolyte.


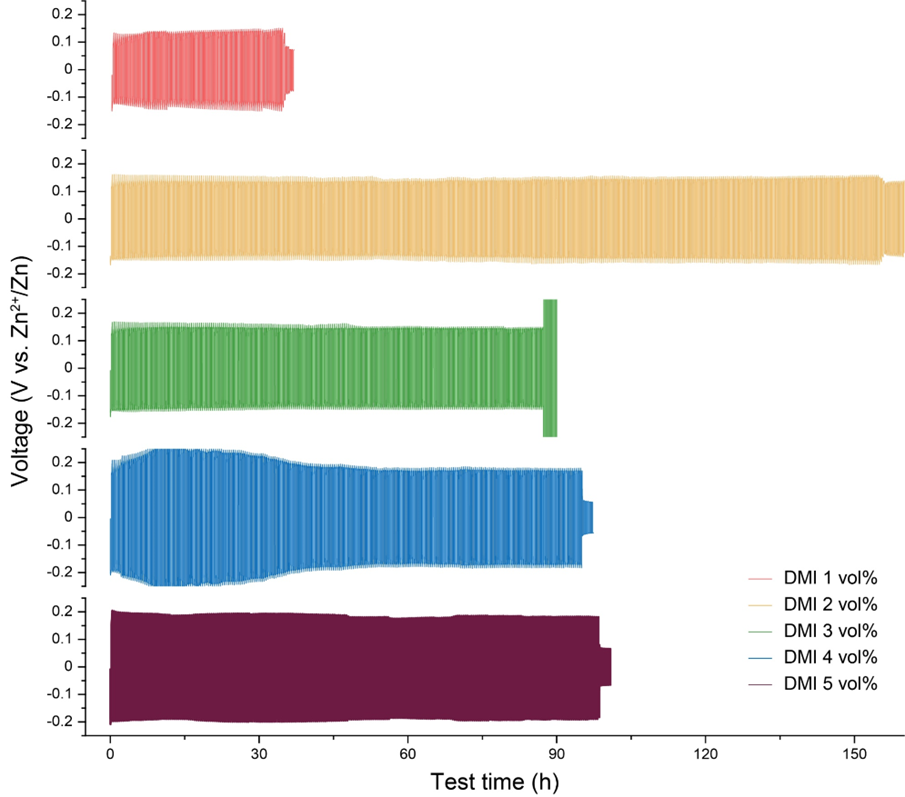


**Figure S4.** Cycling performance of Zn||Zn symmetric cells using different electrolyte operated under 10 mA cm^–2^/2 mAh cm^–2^ operation condition.


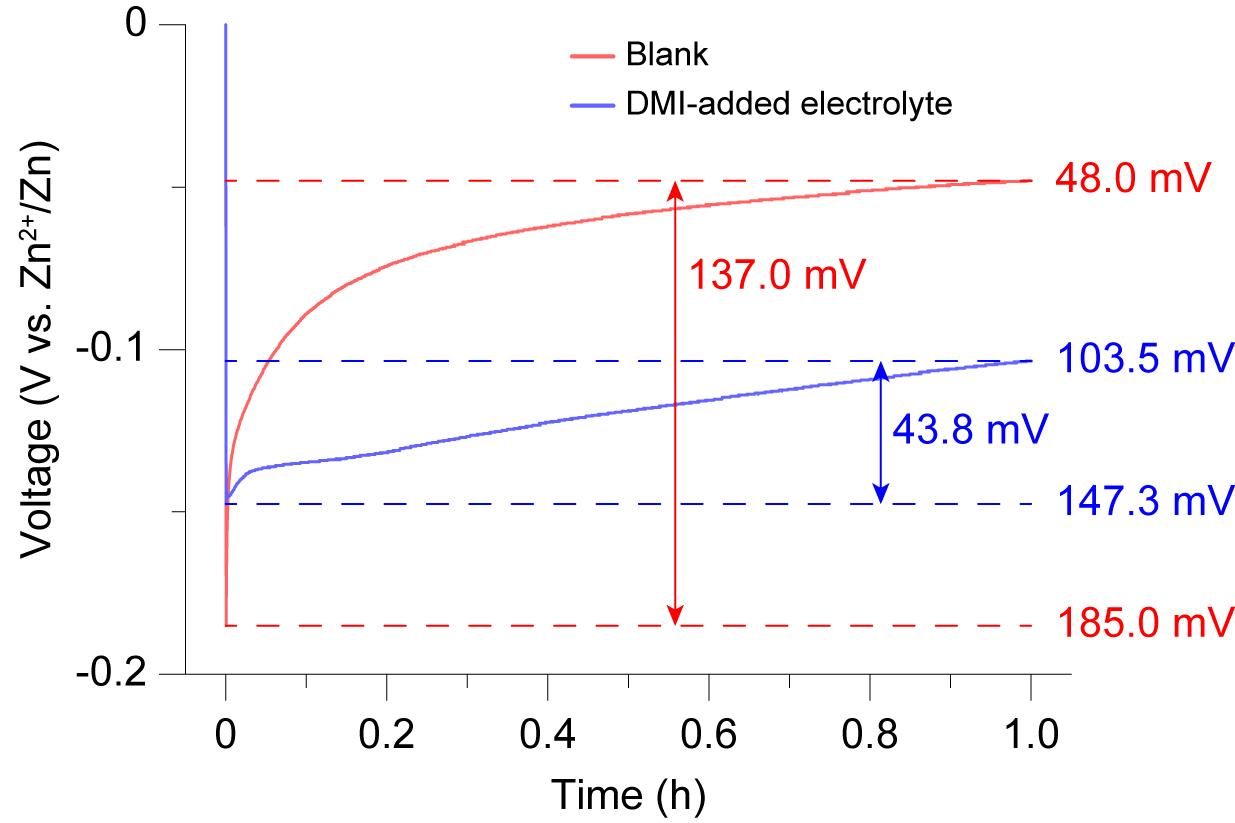


**Figure S5.** Initial zinc deposition overpotential profile in zinc symmetric cell in the blank electrolyte (red) and the DMI-added electrolyte (blue) with the deposition current density of 1 mA cm^–2^.


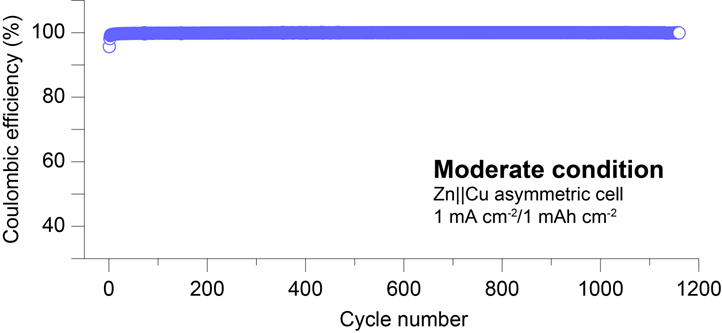


**Figure S6**. Coulombic efficiency of Zn||Cu half-cell with DMI-added electrolyte under 1 mA cm^–2^/1 mAh cm^–2^ operation condition.


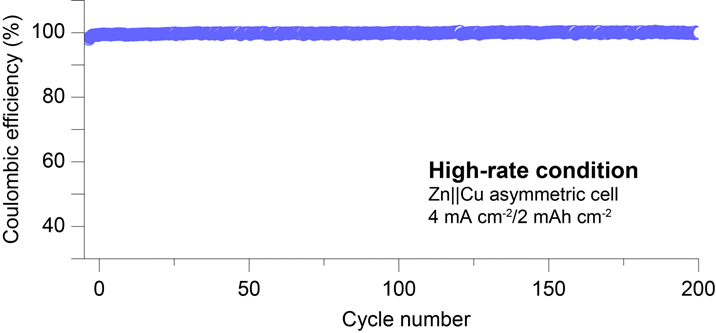


**Figure S7.** Coulombic efficiency of Zn||Cu half-cell with DMI-added electrolyte under 4 mA cm^–2^/2 mAh cm^–2^ operation condition.


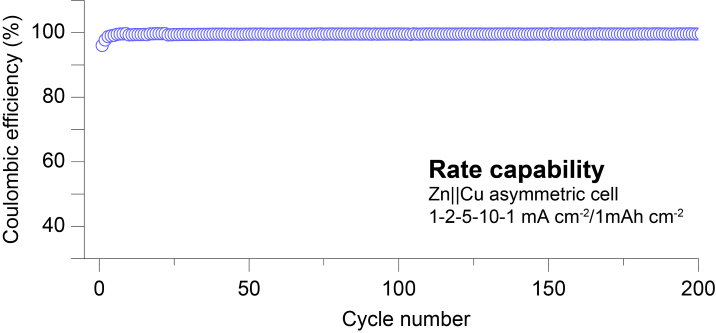


**Figure S8.** Coulombic efficiency of Zn||Cu half-cell with DMI-added electrolyte under rate capability test.


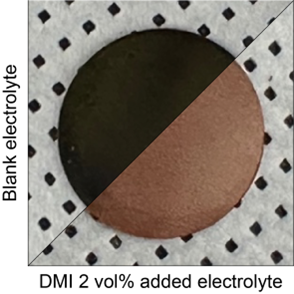


**Figure S9.** Optical image of copper current collectors after immersed in the blank electrolyte and DMI 2 vol% added electrolyte.


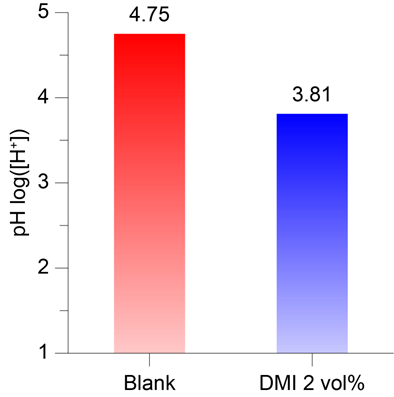


**Figure S10**. The pH value of aqueous electrolytes.


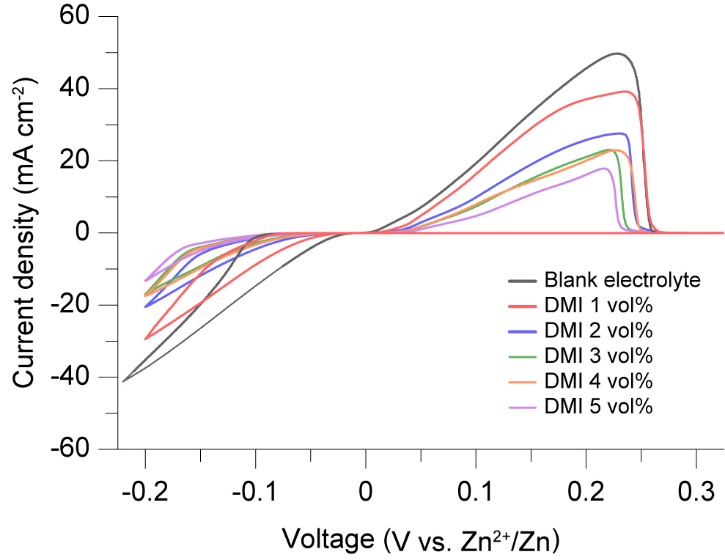


**Figure S11.** Cyclic voltammogram (CV) of the Zn||Cu cells in various DMI content electrolytes.


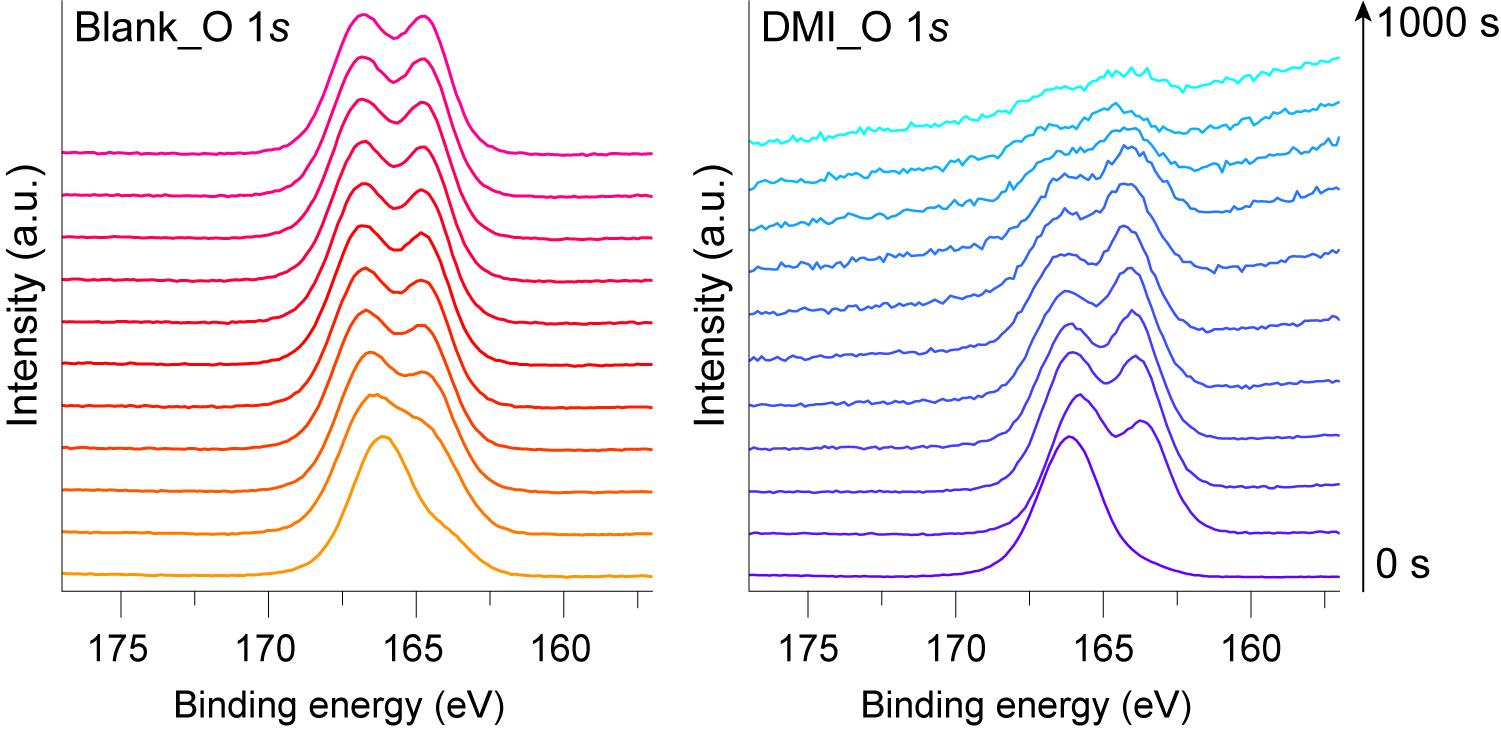


**Figure S12.** XPS depth profile O 1*s* spectra of zinc electrodes immersed in the blank electrolyte and the DMI-added electrolyte.


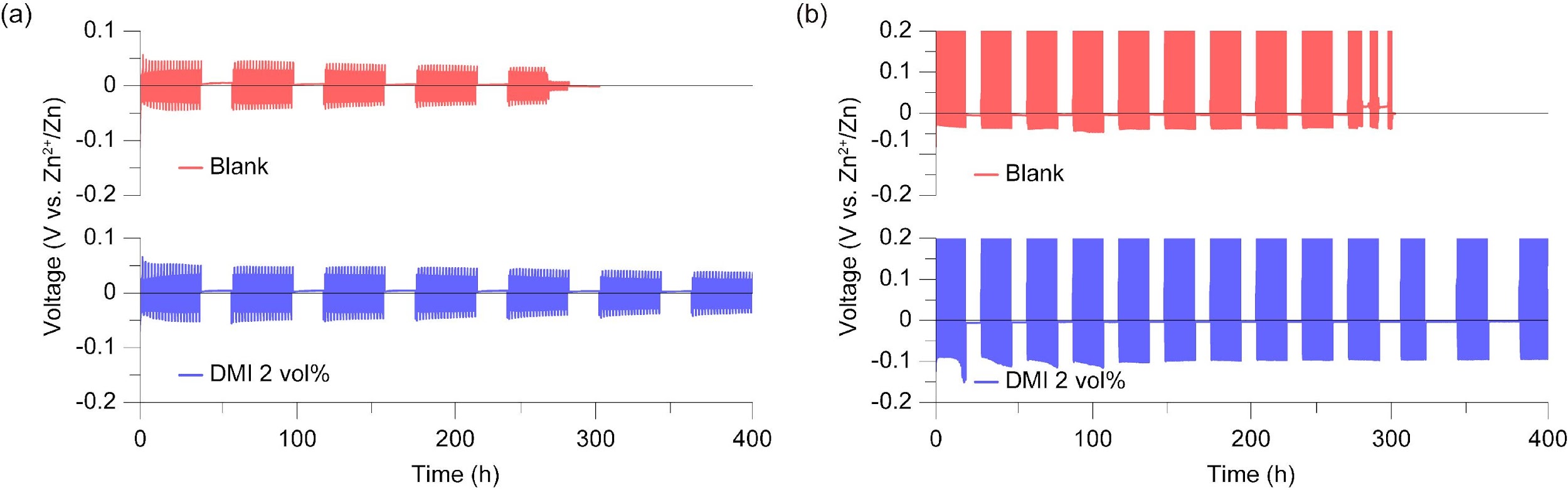


**Figure S13.** Shelving recovery profiles of a) Zn||Zn symmetric cell and b) Zn||Cu asymmetric cell.


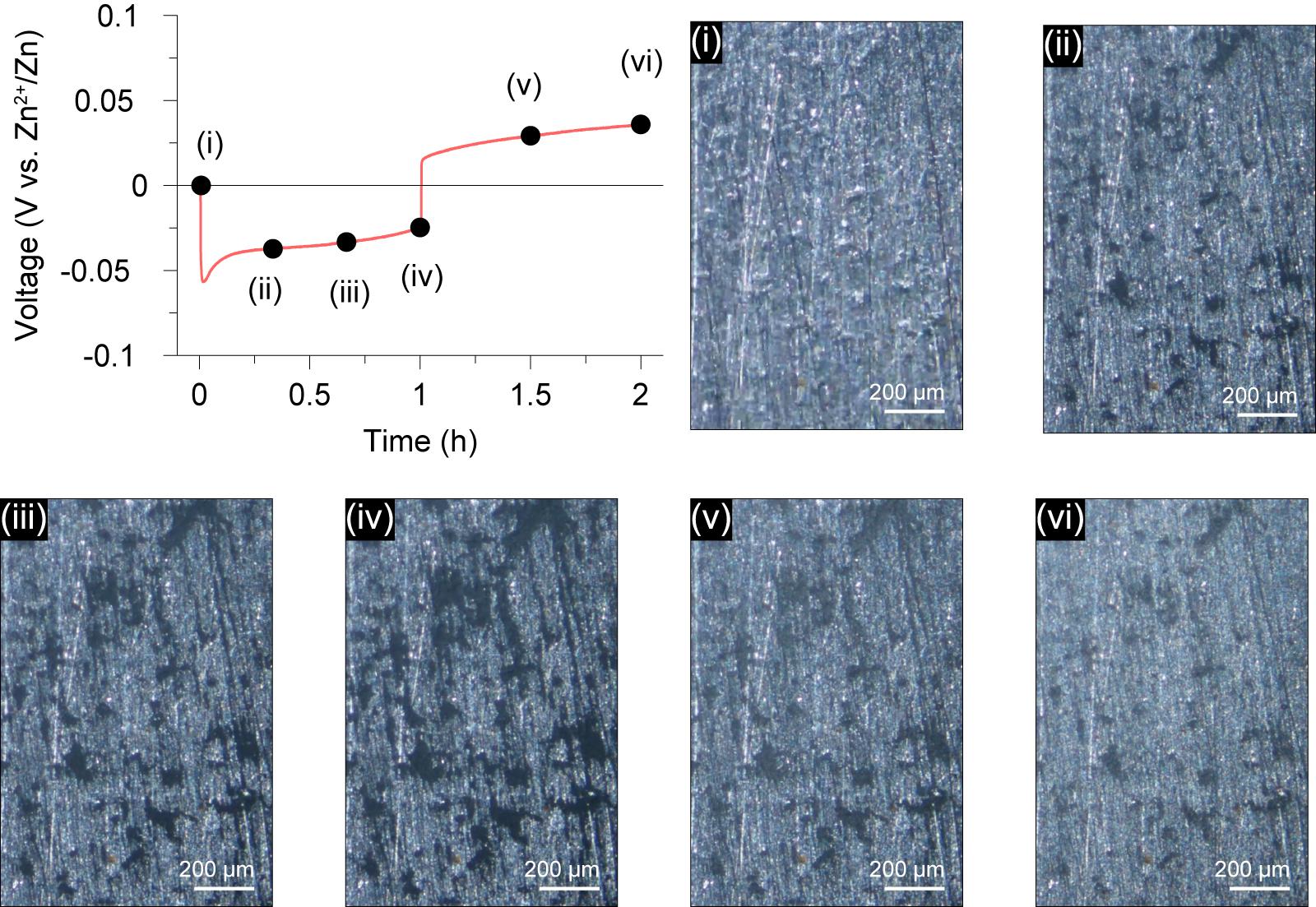


**Figure S14.** Top-view *operando* optical images and voltage profile of the Zn||Zn symmetric cell at 4 mA cm^−2^ and 4 mAh cm^−2^ in the blank electrolyte. Optical images of (i)-(vi) indicate (i) pristine, (ii) 1 h deposition, (iii) 30 min stripping, (iv) 1 h stripping, (v) 30 min re-deposition, and (iv) 1 h re-deposition respectively.


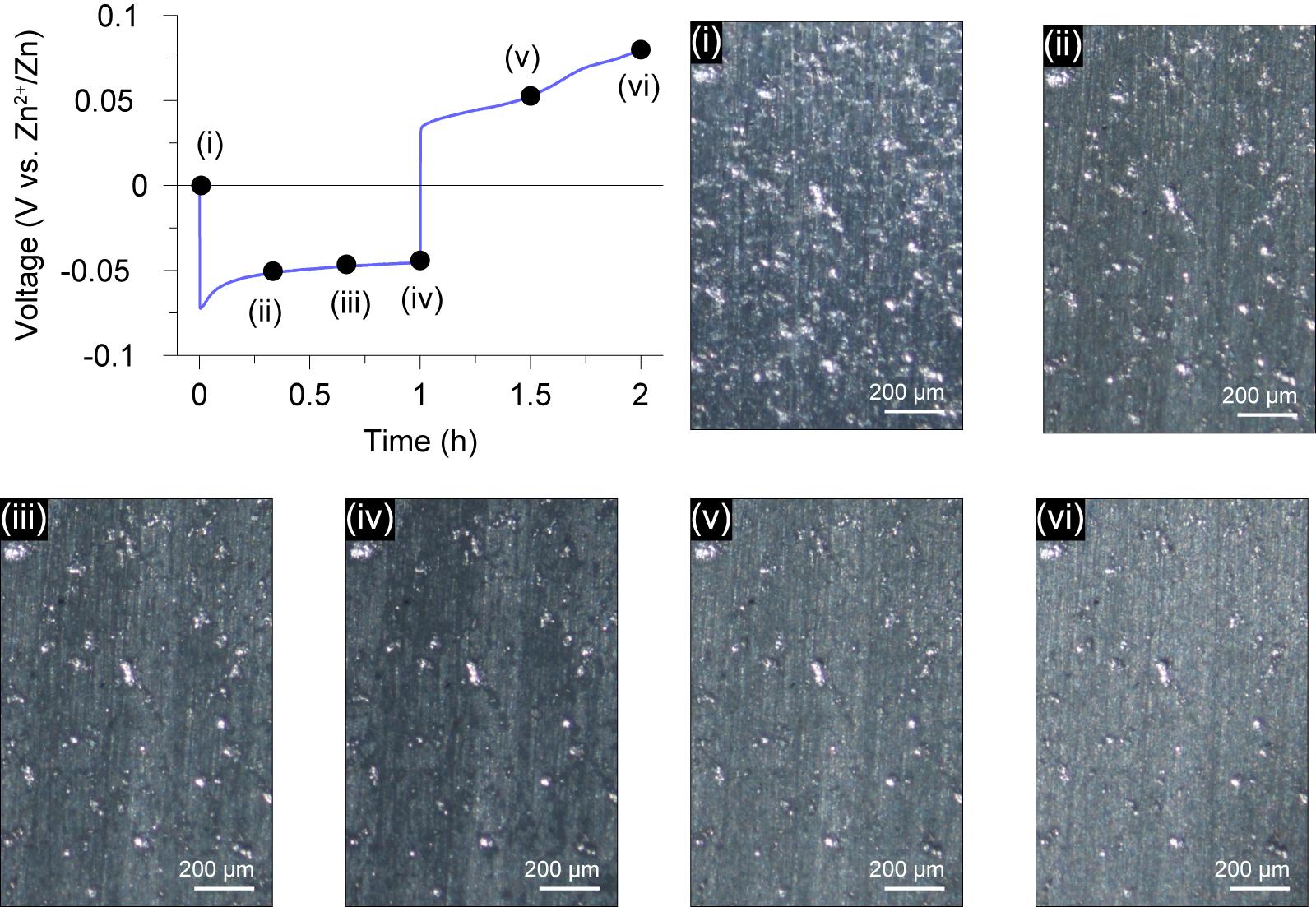


**Figure S15.** Top-view *operando* optical images and voltage profile of the Zn||Zn symmetric cell at 4 mA cm^−2^ and 4 mAh cm^−2^ in the DMI-added electrolyte. Optical images of (i)-(vi) indicate (i) pristine, (ii) 1 h deposition, (iii) 30 min stripping, (iv) 1 h stripping, (v) 30 min re-deposition, and (iv) 1 h re-deposition respectively.


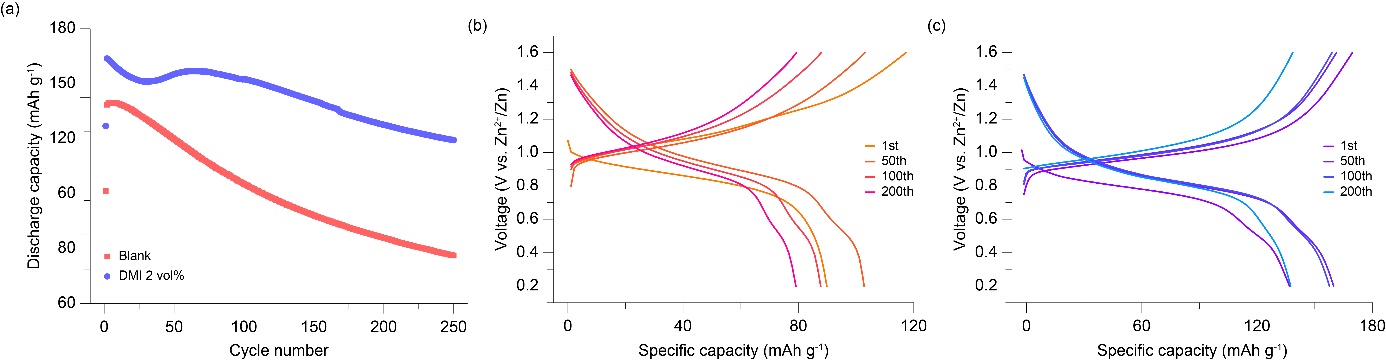


**Figure S16.** a) Cycling stability test of NVO||Zn cells using different electrolytes operated under 2.0 A g^-1^ current density. Voltage profiles of NVO||Zn cell using b) blank electrolyte and c) DMI 2 vol% electrolyte, respectively.
